# Author Correction: An intercross population study reveals genes associated with body size and plumage color in ducks

**DOI:** 10.1038/s41467-018-06521-6

**Published:** 2018-09-25

**Authors:** Zhengkui Zhou, Ming Li, Hong Cheng, Wenlei Fan, Zhengrong Yuan, Qiang Gao, Yaxi Xu, Zhanbao Guo, Yunsheng Zhang, Jian Hu, Hehe Liu, Dapeng Liu, Weihuang Chen, Zhuqing Zheng, Yong Jiang, Zhiguo Wen, Yongming Liu, Hua Chen, Ming Xie, Qi Zhang, Wei Huang, Wen Wang, Shuisheng Hou, Yu Jiang

**Affiliations:** 1 State Key Laboratory of Animal Nutrition; Key Laboratory of Animal (Poultry) Genetics Breeding and Reproduction, Ministry of Agriculture; Institute of Animal Science, Chinese Academy of Agricultural Sciences, Beijing, 100193 China; 2 College of Animal Science and Technology, Northwest A&F University, Yangling, 712100 China; 3 College of Biological Sciences and Technology, Beijing Forestry University, Beijing, 100083 China; 4 Institute of Genetics and Developmental Biology, Chinese Academy of Sciences, Beijing, 100101 China; 5 Beijing Institute of Genomics, Chinese Academy of Sciences, Beijing, 100101 China; 6 Research Center for Ecology and Environmental Sciences, Northwestern Polytechnical University, Xi’an, 710129 China

Correction to: *Nature Communications*; 10.1038/s41467-018-04868-4; published online 17 July 2018

In the original version of this Article, there was an error in the legend for Fig. 2, whereby the descriptions of panels a, b and c were presented in a different order to the corresponding figure panels. The text ‘**a** GWAS of duck plumage color, including 76 colored ducks and 30 white Pekin ducks. The gray horizontal dashed lines indicate the Bonferroni significance threshold of the GWAS (1 × 10^−9^). **b** Fixation index (*F*_ST_) of all SNPs along chromosome 13 between mallards and Pekin ducks. Red dots indicate fixed SNPs. **c** The nucleotide diversity (*π*) of mallards (blue line) and Pekin ducks (red line) from 16.0 to 17.0 Mb on chromosome 13.’ should have read ‘**a** Fixation index (*F*_ST_) of all SNPs along chromosome 13 between mallards and Pekin ducks. Red dots indicate fixed SNPs. **b** The nucleotide diversity (*π*) of mallards (blue line) and Pekin ducks (red line) from 16.0 to 17.0 Mb on chromosome 13. **c** GWAS of duck plumage color, including 76 colored ducks and 30 white Pekin ducks. The gray horizontal dashed lines indicate the Bonferroni significance threshold of the GWAS (1 × 10^−9^).’ This has been corrected in both the PDF and HTML versions of the Article.

