# Supplementary material for: An intercross population study reveals genes associated with body size and plumage color in ducks
Source: Nat Commun. 2018 Jul 17;9:2648. doi: 10.1038/s41467-018-04868-4 (PMC6050300; doi:10.1038/s41467-018-04868-4)
Supplement: Supplementary file 1 — Supplementary Information [file 41467_2018_4868_MOESM1_ESM.pdf]

Supplementary Materials for

Zhou, et al. An intercross population study reveals genes associated with body size and plumage color in ducks

This PDF file includes:

Supplementary Note 1-6

Supplementary Figure 1-16

Supplementary Table 1-22

Supplementary Dataset 1-4

Supplementary Information

CONTENTS

I. List of Supplementary Figures 3

II. List of Supplementary Tables 4

III. List of Supplementary Datasets 6

IV. Supplementary Notes 7

1. F_2 population construction 7

2. The model of ∂a∂i 8

3. Genome scanning for divergent regions 10

4. Melanogenesis pathway gene expression analysis 11

5. *IGF2BP1* differential expression analysis 12

6. Multi-species transcriptome 12

V. Supplementary Figures 14

VI. Supplementary Tables 30

I. List of Supplementary Figures

Supplementary Figure 1. Scatterplot of geographic distance and genetic distance.

Supplementary Figure 2. Diagram of the F_2 population intercross between mallards and Pekin ducks.

Supplementary Figure 3. Comparison of allele frequency spectra (AFSs) between the model and real data for the three duck populations using ∂a∂i.

Supplementary Figure 4. Demographic history of the duck reconstructed from the population resequencing genomes.

Supplementary Figure 5. Allele sharing between Pekin ducks and Gaoyou ducks.

Supplementary Figure 6. Genome-wide admixture analyses inferred from SNPs analyzed in ADMIXTURE 1.3.

Supplementary Figure 7. Genome-wide distribution of the π ln ratio and F_{ST} of 40-kb windows with 10-kb steps across all autosomes.

Supplementary Figure 8. GWAS and linkage analysis of plumage color in F_2-generation ducks.

Supplementary Figure 9. Agarose gel reflecting the genotypes of the indels in *MITF*.

Supplementary Figure 10. The frequency distribution of variation in body size-related traits in the F_2 population.

Supplementary Figure 11. The bin map of the 1026-individual F_2 population from the intercross of mallards and Pekin ducks.

Supplementary Figure 12. Differential gene expression between mallard and Pekin duck at the end of chromosome 28 in multiple tissues.

Supplementary Figure 13. Regional recombination map of 1026 F_2 individuals.

Supplementary Figure 14. Box plots of the phenotype distribution of body size-related traits along the 10 recombination types.

Supplementary Figure 15. Chromosome interaction mapping (Hi-C) and collinearity analysis confirm the scaffold assembly of the end of chromosome 28.

Supplementary Figure 16. The expression pattern of *IGF2BP1* in 5 species.

II. List of Supplementary Tables

Supplementary Table 1. Breed information for mallards, indigenous breeds, and Pekin ducks.

Supplementary Table 2. Summary statistics of the duck genome chromosome-level assembly.

Supplementary Table 3. Mapping details of 106 resequencing samples.

Supplementary Table 4. Distributions of SNPs and indels detected by whole-genome resequencing (106 samples).

Supplementary Table 5. Comparison of three demographic models using fastsimcoal2.

Supplementary Table 6. Parameters and results inferred by ∂a∂i simulations.

Supplementary Table 7. D-statistics of D (Muscovy duck (W), Pekin duck (X); GY (Y), Z).

Supplementary Table 8. The mean and threshold of the F_{ST} and π ln ratio.

Supplementary Table 9. Genomic regions identified as CDRs between mallard and indigenous ducks.

Supplementary Table 10. Genomic regions identified as candidate divergence regions between indigenous ducks and Pekin ducks.

Supplementary Table 11. The CDRs that undergo continuous selection with the same trend in frequency change in the domestication (dom) and improvement (imp) stages.

Supplementary Table 12. Fixed SNPs (F_{ST} = 1) along the duck genome.

Supplementary Table 13. Genotype frequency for variants with F_{ST} = 1 along the duck genome in additional samples.

Supplementary Table 14. Primers used in the experiment in relation to the two critical genes *MITF* and *IGF2BP1*.

Supplementary Table 15. The top 10 SNPs for body size-related traits in the genome-wide association study based on the F_2 population of 1026.

Supplementary Table 16. The top loci of body size-related traits in the genome-wide linkage analysis based on the F_2 population of 1026.

Supplementary Table 17. Statistics for synonymous and missense variants at the end

of chromosome 28 (4,400,000 bp-4,710,000 bp).

Supplementary Table 18. Expression levels of 19 protein-coding genes at the end of chromosome 28 in multiple tissues of mallards and Pekin ducks.

Supplementary Table 19. Expression level of *IGF2BP1* in multiple tissues of mallards and Pekin ducks. Tissues from 1-day-old (1D), 2-week-old (2W), 4-week-old (4W) and 8-week-old (8W) ducks were sampled, and the gene expression levels were measured by qPCR.

Supplementary Table 20. Expression levels of *IGF2BP1* in different recombinant types of F_2 individuals. The gene expression levels were measured by qPCR.

Supplementary Table 21. Summary of Hi-C data in duck.

Supplementary Table 22. *IGF2BP1* expression levels in (a) human, (b) mouse, (c) chicken, and (d) zebrafish.

III. List of Supplementary Datasets

Supplementary Dataset 1. The duck variation map.

Supplementary Dataset 2. The ancestral state of duck SNPs.

Supplementary Dataset 3. Global transcriptional data for mallards and Pekin ducks.

Supplementary Dataset 4. The significant loci in the genome-wide association study of the F_2 population.

Supplementary Datasets 1 to 4 are available at www.figshare.com/projects/Duck_Project/24214 or www.duckbase.org/Download.

IV. Supplementary Notes

1. F_2 population construction

The F_2 segregating population is the most informative population for genetic analysis. In addition, it may have considerable utility in QTL mapping and identifying the loci for economically important traits. In this study, the experimental population was derived from a cross between mallards and Pekin ducks. The features of mallards include excellent flying ability and a relatively slow growth rate. The Pekin duck is a world-famous domestic duck breed known for its extraordinary body size and fast growth rate. Adult Pekin ducks reach an average weight of 3.5 kilograms. Domesticated ducks differ from their wild mallard progenitor in a wide array of morphological and physiological traits.

We constructed the duck F_2 population starting in 2014 at the duck farm of the Institute of Animal Science, Chinese Academy of Agricultural Sciences (**Supplementary Fig. 2**). The animals' care was in accordance with institutional guidelines. To our knowledge, this population is the largest duck population worldwide. The F_1 generation was produced from reciprocal crosses of mallards and Pekin ducks. In an orthogonal experiment, 100 mallards were selected as the female parents and 10 Pekin ducks were selected as the male parents. In a backcross, 4 male mallards and 40 female Pekin ducks were the male and female parents, respectively. The size of the F_1 generation reached more than 700 individuals, and the ratio of male to female ducks was relatively balanced. The F_1 offspring, with distinctly different parental types, displayed a new, uniform phenotype with a combination of characteristics from the parents. Although genetic performances differed within the generation of reciprocal crosses to a certain extent, the F_1 generation was generally heterozygous and consistent. For example, all F_1 hybrids had both white feathers and mottled or black feathers.

The F_2 generation was produced from the natural mating of F_1 hybrids, and the mating was internally limited to the orthogonal and backcross experiments. While building the families, we considered and complied with the following principles: (1) the ratio of males to females was one to three; (2) males and females in the same family were

not from the same nest; and (3) female ducks within a family were not half-siblings. This mating system allows abundant genetic diversity. In total, 216 families were established using the F_1 hybrids. The F_2 generation was composed of almost 2000 ducks that displayed segregation of various genetic characteristics, including feather color and body weight.

When the ducklings hatched, we recorded their feather colors and acquired several photos of each individual. Moreover, the ducks were weighed each week after hatching. At three weeks of age, part of the F_2 generation (500 individuals) was moved from the duckling house to the shed designed to measure feed efficiency. The remaining ducks were housed indoors or outdoors and raised to the age of eight weeks. We performed a slaughter experiment of more than 1000 ducks and measured a series of traits, including appearance, carcass characteristics, blood biochemical indices, and quality of meat. The remaining ducks were sequentially raised to study reproductive performance, egg quality and eggshell properties. Furthermore, samples of blood from all F_0, F_1 and F_2 ducks were obtained for DNA extraction and biochemical examination. Tissues were sampled for RNA or protein extraction and used in the transcriptome and proteome analyses.

2. The model of ∂a∂i

For the three duck populations (mallards (Mal), indigenous breeds (Ind) and Pekin ducks (Pek)), we constructed three possible models:

(1) (Mal, (Ind, Pek)), in which ducks were first split into Mal and non-Mal populations and then the non-Mal group was split into Ind and Pek populations;

(2) (Ind, (Mal, Pek)); and

(3) (Pek, (Mal, Ind)), which is similar to the first model.

We started simulations with a null model to identify the most suitable model for our populations using fastsimcoal2’s Akaike information criterion (AIC method). As shown in **Supplementary Table 4**, the first model had the highest log-likelihood and AIC values; therefore, it was chosen as the best model of duck phylogeny. Then, we increased the model complexity by gradually adding parameters. We performed the

subsequent simulation using a ∂a∂i approach.

The Python function used to estimate the final model parameters in ∂a∂i is as follows:

```
def ne_domestication_event((Ta,Tb,nu_dom,nu1,nu2_0,nu2,nu3_0,nu3,m1,m2), (n1,n2,n3), pts):
    # Define the grid that we will use
xx=Numerics.default_grid(pts)
# phi for the equilibrium ancestral population
phi=PhiManip.phi_1D(xx)
# model the population1 split at time Ta with no migration event and new population sizes of nu1 and nu_dom.
    phi=PhiManip.phi_1D_to_2D(xx,phi)
    phi=Integration.two_pops(phi,xx,Ta,nu1=nu1,nu2=nu_dom,m12=0,m21=0)
   # model the population2 and population3 split at time Tb with new population sizes of nu2_0 and nu3_0, size growth within the time, and migration between the two populations
    phi=PhiManip.phi_2D_to_3D_split_2(xx, phi)
    nu2_func=lambda t: nu2_0*(nu2/nu2_0)**(t/Tb)
nu3_func=lambda t: nu3_0*(nu3/nu3_0)**(t/Tb)
#model recent population size change at time Tb with intensity nu1, nu2_func, nu3_func
phi=Integration.three_pops(phi,xx,Tb,nu1=nu1,nu2=nu2_func,nu3=nu3_func,m12=0, m21=0,m13=0,m31=0,m23=m1,m32=m2)
    fs=Spectrum.from_phi(phi, (n1,n2,n3), (xx,xx,xx))
    return fs
```

The simulation results of the model are shown in **Supplementary Fig. 4** and **Supplementary Table 5**.

3. Genome scanning for divergent regions

Selection of beneficial mutations affects the patterns of genetic variation at surrounding loci, causing (i) a reduction in heterozygosity, (ii) a skewed allele frequency distribution, and (iii) an excess of high-frequency derived alleles near the selected allele[1]. We detected the candidate divergent regions (CDRs) by searching the genome for regions with high fixation index (F_{ST}) values[2] and high differences in genetic diversity (π ln ratio).

The major challenge in such an analysis is to distinguish true sweeps from fixation due to genetic drift. We addressed this problem by sampling different populations. For instance, mallards were sampled from different places in different years, indigenous breeds were sampled from 12 populations, and Pekin ducks were drawn from 3 different populations. Since the variance of F_{ST} depends on the number of SNPs used for each calculation, spurious selection signals are most likely in windows with few variable sites. We reduced the number of false positives by excluding windows with fewer than 40 variable sites from the analysis. Even if the windows harbor sufficient numbers of variable sites, it can still be challenging to differentiate signals caused by selection from genetic drift. To address this issue, two parameters (window size and threshold) can be tuned to reduce the risk of higher false positives caused by drift. However, determining an optimal window size, in terms of maximizing the sensitivity to detect selection events at a low cost of false positives, is complicated given the complex and partly unknown demographic history of the duck. It is well established that selection can impact most loci linked to selected loci and can make neutral loci display selective features. If it is known that most loci in windows are within an LD region, we can maximize the window to balance the false positives and false negatives. Therefore, we chose half the LD decay of the Pekin duck (approximately 40 kb) as the window size. Similarly, it is difficult to assign strict thresholds that distinguish selection and drift. Here, we focused our description of putatively selected regions on the windows with a significance level of $P < 0.005$ (Z test) in both the F_{ST} and π ln ratios, as these windows represented the extreme ends of the distributions (**Supplementary Fig. 7** and **Supplementary Table 7**). We further verified whether

this threshold was appropriate by performing permutations in F_{ST} analysis. Because sex chromosomes differ from autosomes in several properties that can affect population genetic estimates (such as effective population size, mutation rate, and recombination rate), we partitioned the genome into autosomes and sex chromosomes to detect outlier windows. We first mixed the samples of two populations together and then randomly divided them into two new populations. Next, we calculated windowed F_{ST} values between these two new populations (in sliding 40-kb windows with 10-kb steps) and filtered out the windows with fewer than 40 SNPs. Subsequently, we recorded the maximum value across all windows and repeated this process 100 times. Finally, we sorted the 100 maximum values from largest to smallest and selected the fifth value as the final permutation result (**Supplementary Fig. 7** and **Supplementary Table 7**). We found that the threshold defined by the Z test was much greater than the threshold of permutations in each pairwise comparison. This result indicates that using $P < 0.005$ (Z test) as a threshold can effectively exclude false positives. However, it is possible that windows with lower F_{ST} may also merit further investigation, as such windows may have also contributed to duck domestication.

4. Melanogenesis pathway gene expression analysis

F_{ST} analysis indicated that the *MITF* gene loci were intensively selected during domestication. A GWAS revealed that *MITF* loci had a strong association with feather color. To explore whether the expression of genes related to plumage color was upregulated or downregulated, we examined RNA-seq data for feather follicles to examine the gene expression of *MITF* and other genes in the melanogenesis pathway (http://www.genome.jp/kegg-bin/show_pathway?apla04916).

Differential gene expression between mallards and Pekin ducks was inferred using the R/Bioconductor package edgeR [3]. Before samples with different library sizes and RNA composition were compared, normalization was conducted using the "calcNormFactors()" function. The default method for computing these scale factors uses a trimmed mean of M-values (TMM) between each pair of samples. We then estimated the quantile-adjusted conditional maximum likelihood (qCML) common

dispersion using the estimateCommonDisp() function and the qCML tagwise dispersions using the estimateTagwiseDisp(). The top differentially expressed tags have very small p-values and FDR values, as well as large fold changes. The edgeR results for feather follicles showed that (1) *MITF* itself did not show differential expression between Pekin duck and mallard; (2) downstream genes, including *MLANA, TYR, TYRP1, DCT, OCA2,* and *MLPH*, were significantly downregulated (**Supplementary Dataset 3**).

5. *IGF2BP1* differential expression analysis

Nineteen coding genes reside at the end of chromosome 28, which showed a high F_{ST} value, making it difficult to distinguish the critical gene under strong selection. Therefore, we explored the expression levels of 19 protein-coding genes in this region. Through the edgeR analysis described above, we found that only *IGF2BP1* specifically showed significant differential expression both spatially and temporally between mallards and Pekin ducks, for example, in skin, abdominal adipose tissue, liver, and thigh cartilage at 1 day, 2 weeks, 4 weeks, and 8 weeks. This result provides strong evidence that *IGF2BP1* is the critical selected gene at the end of chromosome 28 (**Supplementary Fig. 12** and **Supplementary Table 19**).

6. Multispecies transcriptional profiling of *IGF2BP1*

To further explore the expression pattern of *IGF2BP1*, we downloaded the expression data for human, mouse, zebrafish, and chicken based on public transcriptome data from the NCBI website (**Supplementary Table 20**). We directly obtained the human and mouse FPKM values of *IGF2BP1* from the website. Moreover, we downloaded the SRA files of zebrafish and chicken and converted them into FASTQ format using the SRA toolkit. We then aligned the short reads to their reference genome using TopHat2. Subsequently, BAM files were sorted by samtools[4], and FPKM values were calculated by Cufflinks[5].

Supplementary References

1. Stephan, W. Genetic hitchhiking versus background selection: the controversy and its implications. *Philos. Trans. R. Soc. Lond. B. Biol. Sci.* **365**, 1245-53 (2010).
2. Weir, B.S. & Cockerham, C.C. Estimating F-Statistics for the Analysis of Population-Structure. *Evolution* **38**, 1358-1370 (1984).
3. Robinson, M.D., McCarthy, D.J. & Smyth, G.K. edgeR: a Bioconductor package for differential expression analysis of digital gene expression data. *Bioinformatics* **26**, 139-140 (2010).
4. Li, H. *et al.* and 1000 Genome Project Data Processing Subgroup, The sequence alignment/map format and SAMtools. *Bioinformatics* **25**, 2078-9 (2009).
5. Trapnell, C. *et al.* Transcript assembly and quantification by RNA-Seq reveals unannotated transcripts and isoform switching during cell differentiation. *Nat. Biotechnol.* **28**, 511-5 (2010).

Supplementary Figures

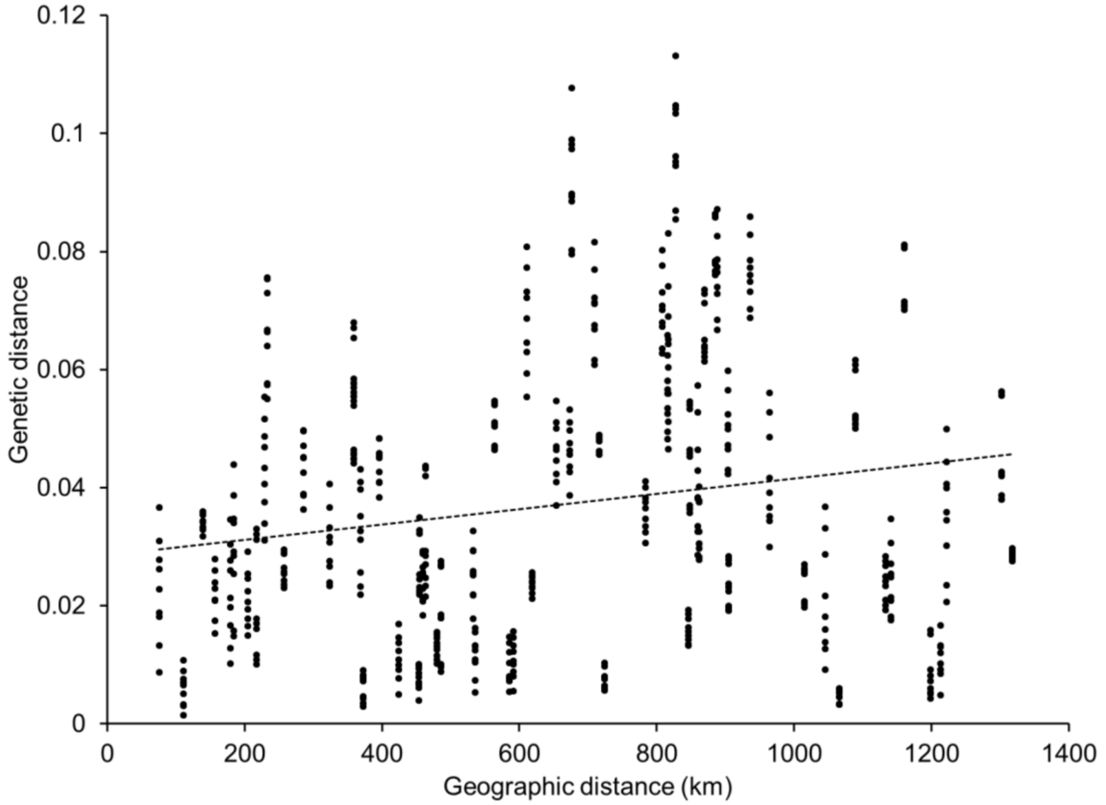

Supplementary Figure 1. Scatterplot of geographic distance and genetic distance. Comparisons within indigenous duck breeds are represented by black dots. The dashed regression line was fitted to the data ($D_{genetic} = 0.0285 + (1 \times 10^{-5}) \times D_{geographic}$, $R^2 = 0.0343$).

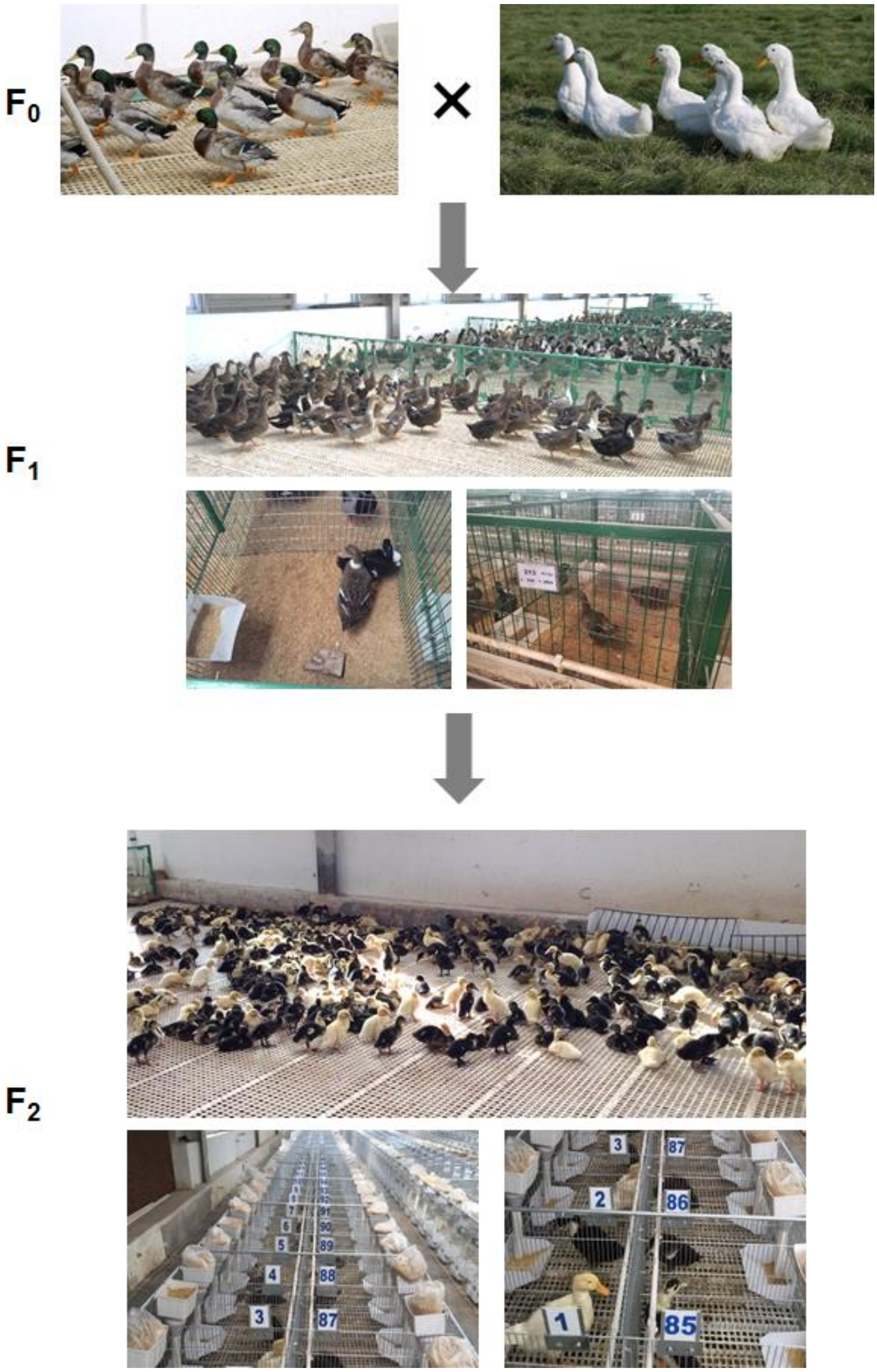

Supplementary Figure 2. Diagram of the F_2 population intercross between mallards and Pekin ducks. The facility crossover cage and feed efficiency measurement cage are shown below the F_1- and F_2-generation populations.

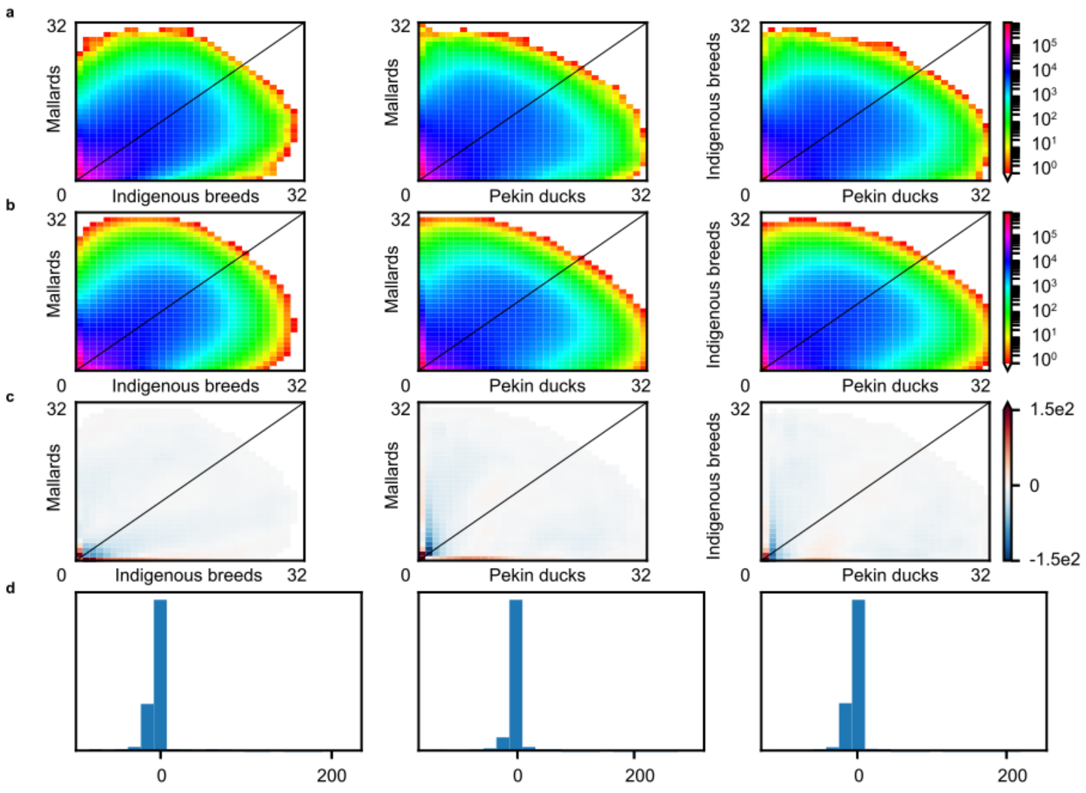

Supplementary Figure 3. Comparison of allele frequency spectra (AFSs) between the model and real data for the three duck populations using ∂a∂i. (**a**) Marginal AFS of the real data for each pair of populations (mallards/indigenous breeds, mallards/Pekin ducks, indigenous breeds/Pekin ducks). (**b**) AFS of the maximum-likelihood model simulated based on the real data. The residuals between the model and real data are shown in (**c**) heat maps and (**d**) bar graphs.

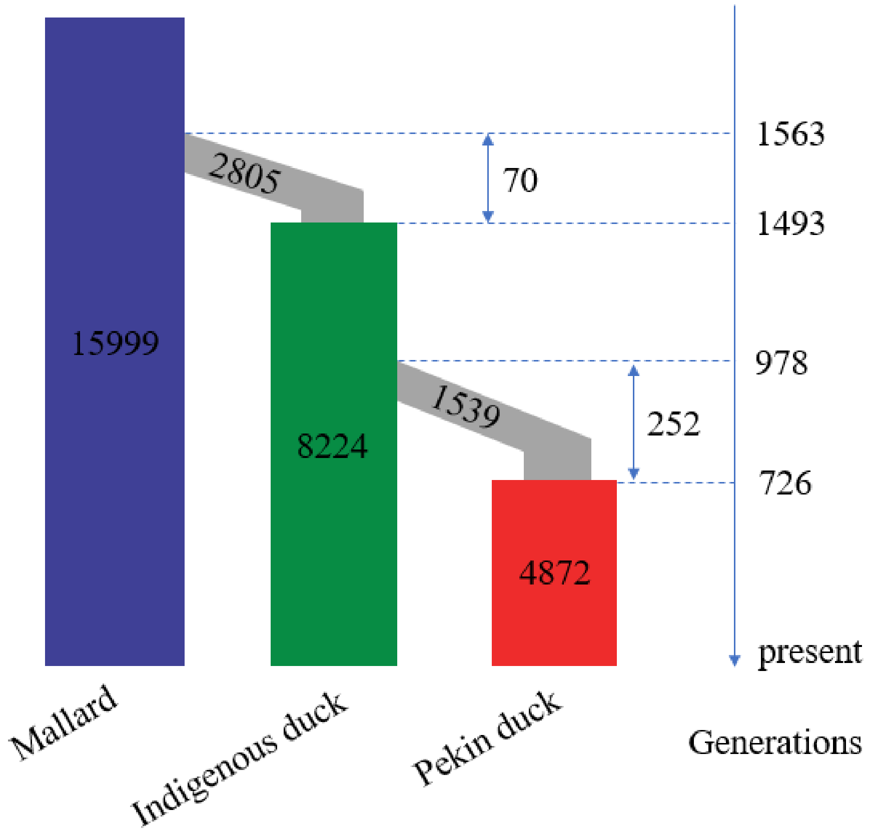

Supplementary Figure 4. Demographic history of the duck reconstructed from the population resequencing genomes. The ∂a∂i results show that the demographic history of the duck spans from ~1,563 generations ago to the present, with a mutation rate of $\mu = 2.3 \times 10^{-9}$. The numbers in all boxes show changes in the effective population size. Mallards and indigenous breeds split off from each other 1563 generations ago, and Pekin ducks split off from indigenous breeds 978 generations ago.

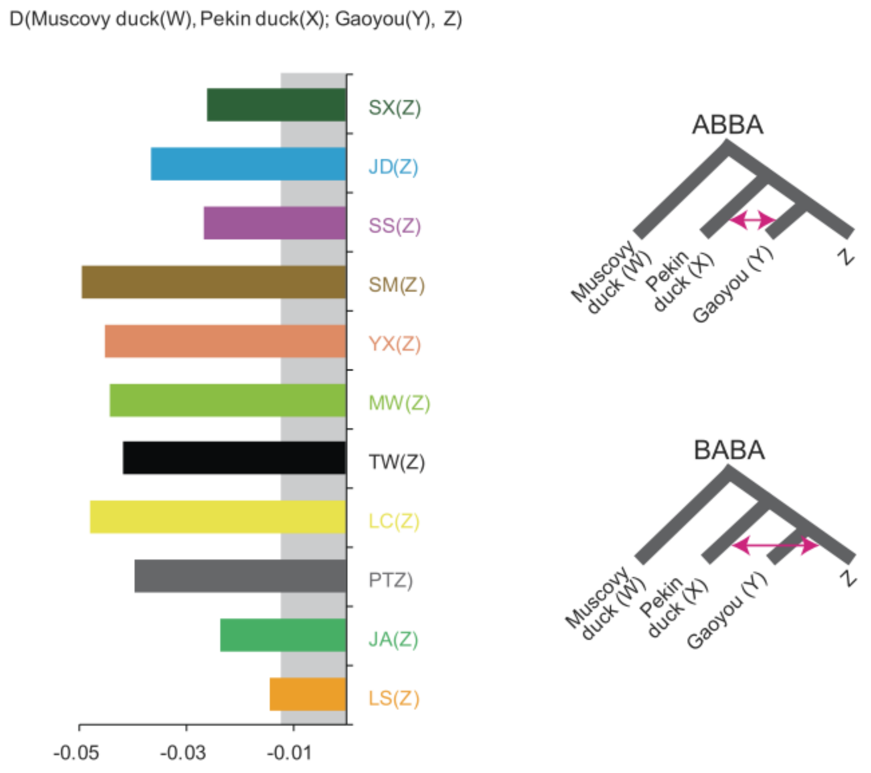

Supplementary Figure 5. Allele sharing between Pekin ducks and Gaoyou ducks. The results were quantified by D-statistics (Supplementary Table 6). A positive D-statistic value indicates that the X and Z populations share more derived alleles than X and Y share, while a negative value indicates that the X and Y populations share more derived alleles than X and Z share. The shaded area delimits the confidence interval, defined by |Z -score| ≤ 3.

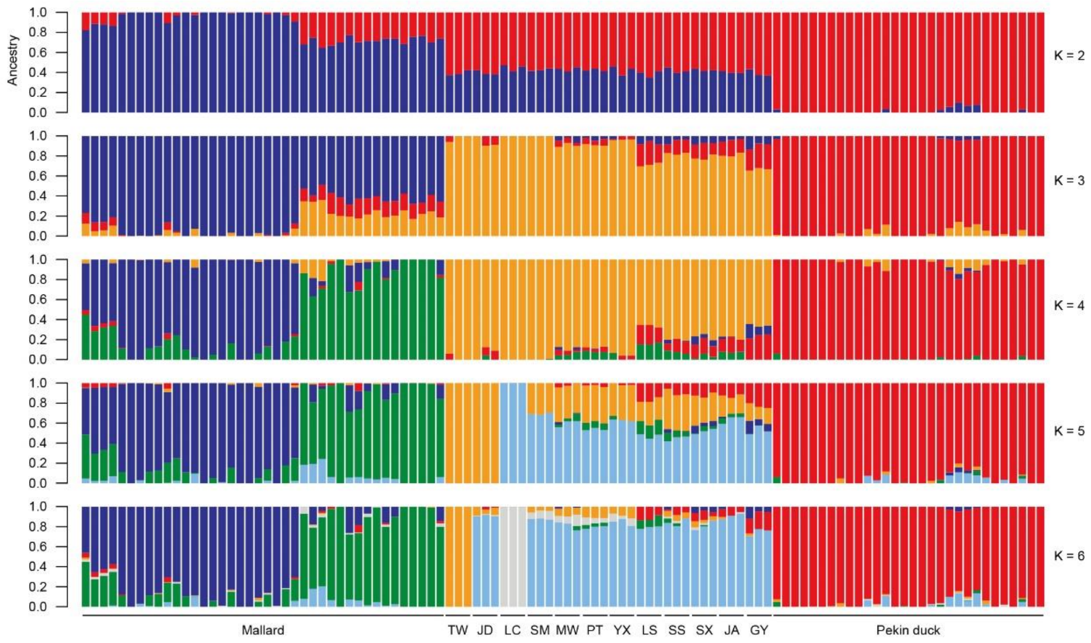

Supplementary Figure 6. Genome-wide admixture analyses inferred from SNPs analyzed in ADMIXTURE 1.3. At K = 3, three populations – mallards, indigenous breeds, and Pekin ducks – separate distinctively, consistent with the results of the phylogenetic tree. At K = 4, 5, and 6, subpopulations among mallards and some indigenous breeds, especially the Gaoyou (GY) duck, share some of their composition with Pekin ducks. This result indicates a potential slight gene flow between Pekin ducks and indigenous breeds.

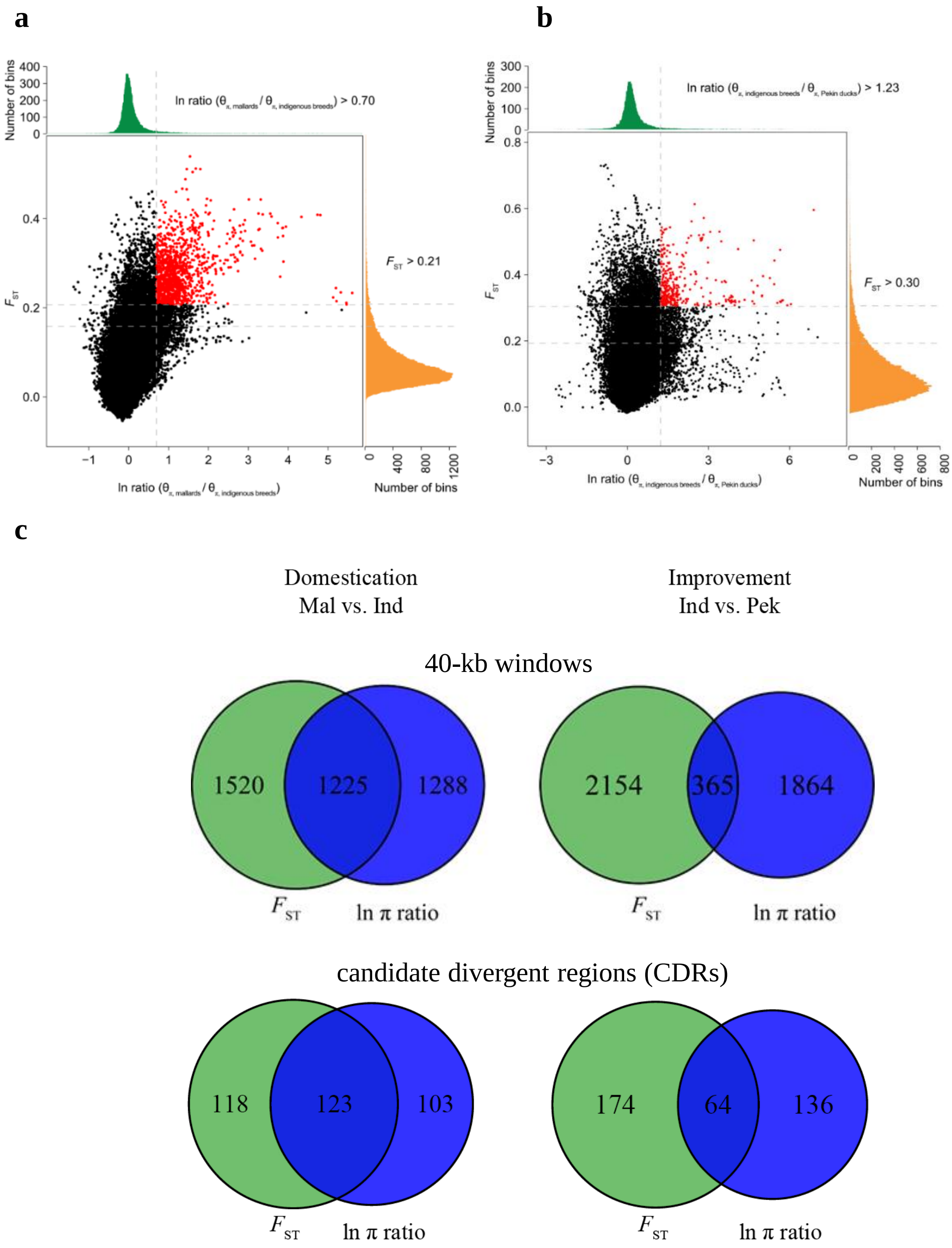

Supplementary Figure 7. Genome-wide distribution of the π ln ratio and F_{ST} of 40-kb windows with 10-kb steps across all autosomes. (**a**) Between mallards and indigenous breeds and (**b**) between indigenous breeds and Pekin ducks. Red dots represent windows fulfilling the requirement for selected regions (corresponding to Z test $P < 0.005$). The gray vertical dashed line shows the threshold of the π ln ratio, and the two gray horizontal dashed lines show the threshold of F_{ST} and the permutation threshold with 100 bootstraps. (**c**) The overlapped sweep windows (40-kb) and CDRs of F_{ST} and π ln ratio the during domestication and improvement stages.

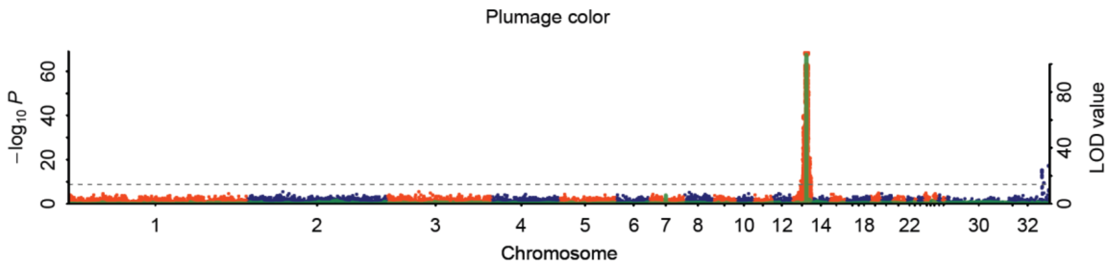

Supplementary Figure 8. GWAS and linkage analysis of plumage color in F_2-generation ducks. All the F_2 individuals were classified into two categories: colored and white. The green line indicates the LOD values, and the gray horizontal dashed line indicates the Bonferroni significance threshold of the GWAS (1×10^{-9}).

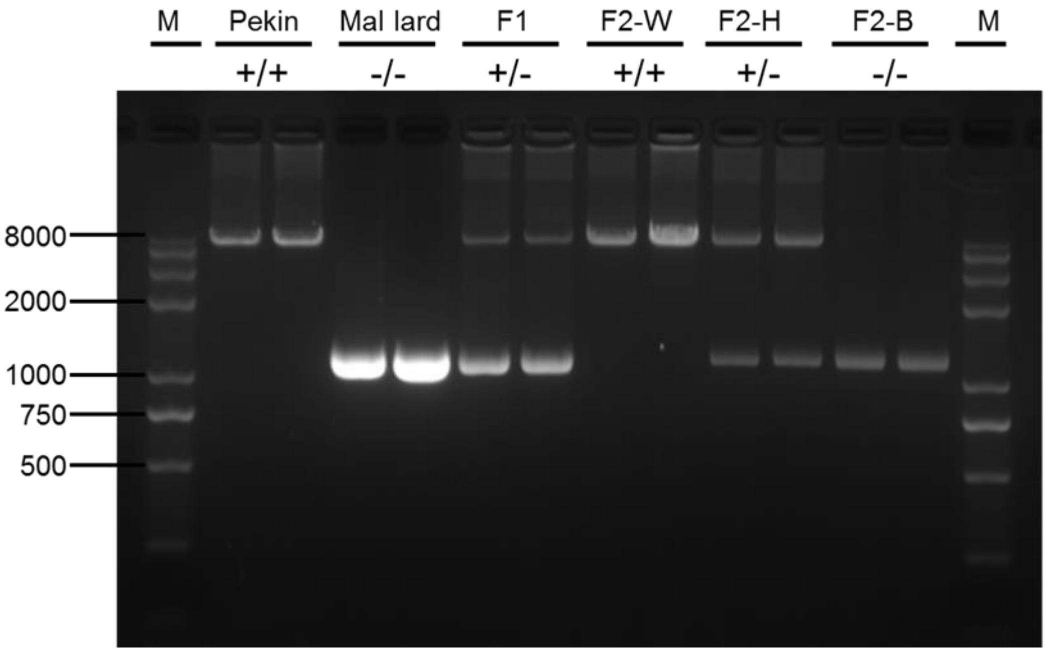

Supplementary Figure 9. Agarose gel reflecting the genotypes of the indels in *MITF*. +/+ represents a homozygous insertion, -/- a homozygous deletion, and +/- a heterozygous genotype. We designed primers using the sequence flanking the insertion in Pekin ducks and performed PCR experiments using ducks from the F_2 segregating population. M represents the DNA marker; Pekin, Pekin ducks; and F_1, F_1 hybrids from reciprocal crosses between Pekin ducks and mallards. The genotypes of Pekin ducks, mallards, and F_1 hybrids are homozygous insertion, homozygous deletion, and heterozygous, respectively. For the F_2 generation, we used F_2-W, F_2-H, and F_2-B to represent white, white along with black (or black along with white), and entirely black feathers, respectively. The three phenotypes all appeared in the F_2 generation, as shown in **Fig. 3b**.

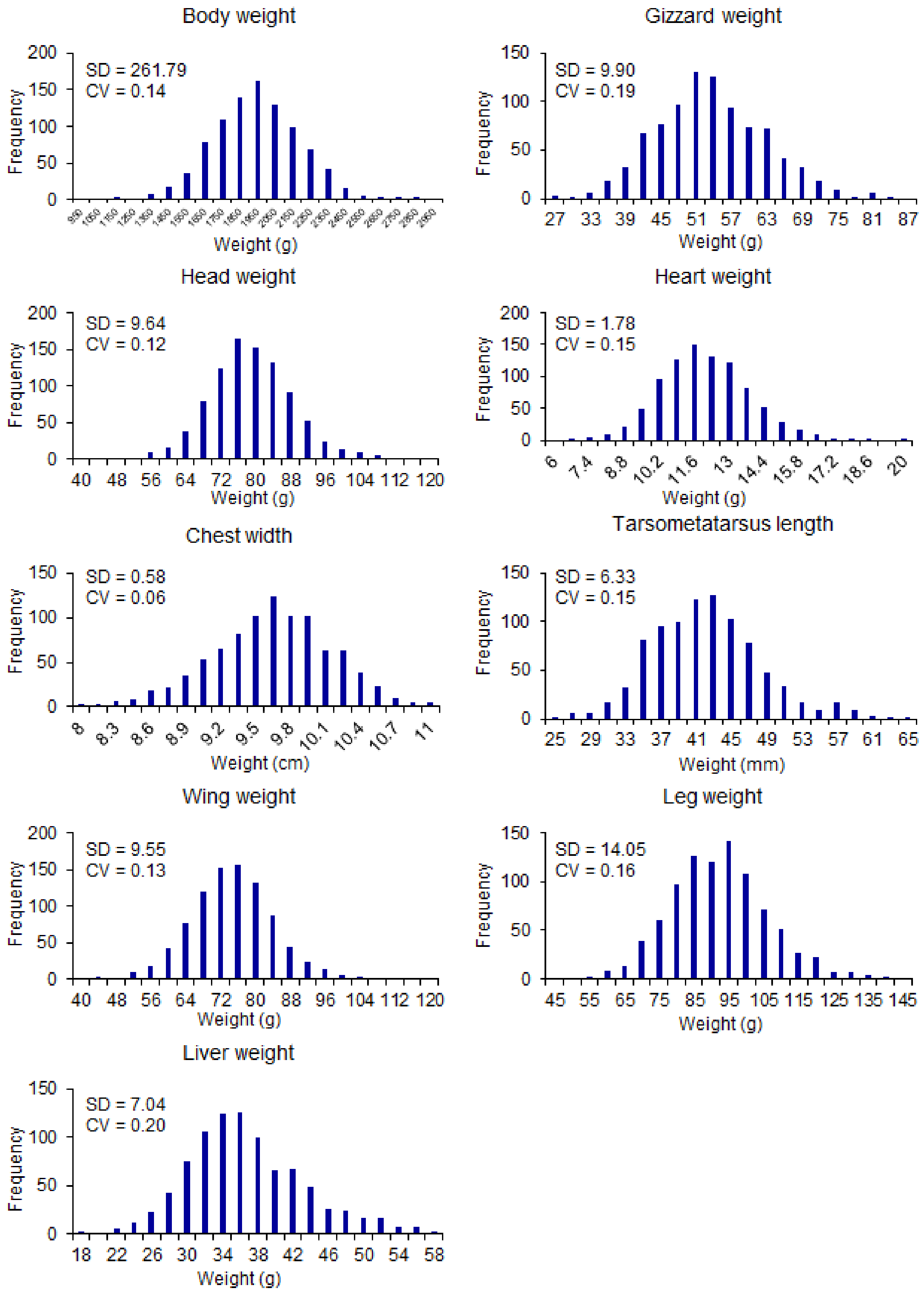

Supplementary Figure 10. The frequency distribution of variation in body size-related traits in the F_2 population. SD and CV are the abbreviations for standard deviation and coefficient of variation, respectively.

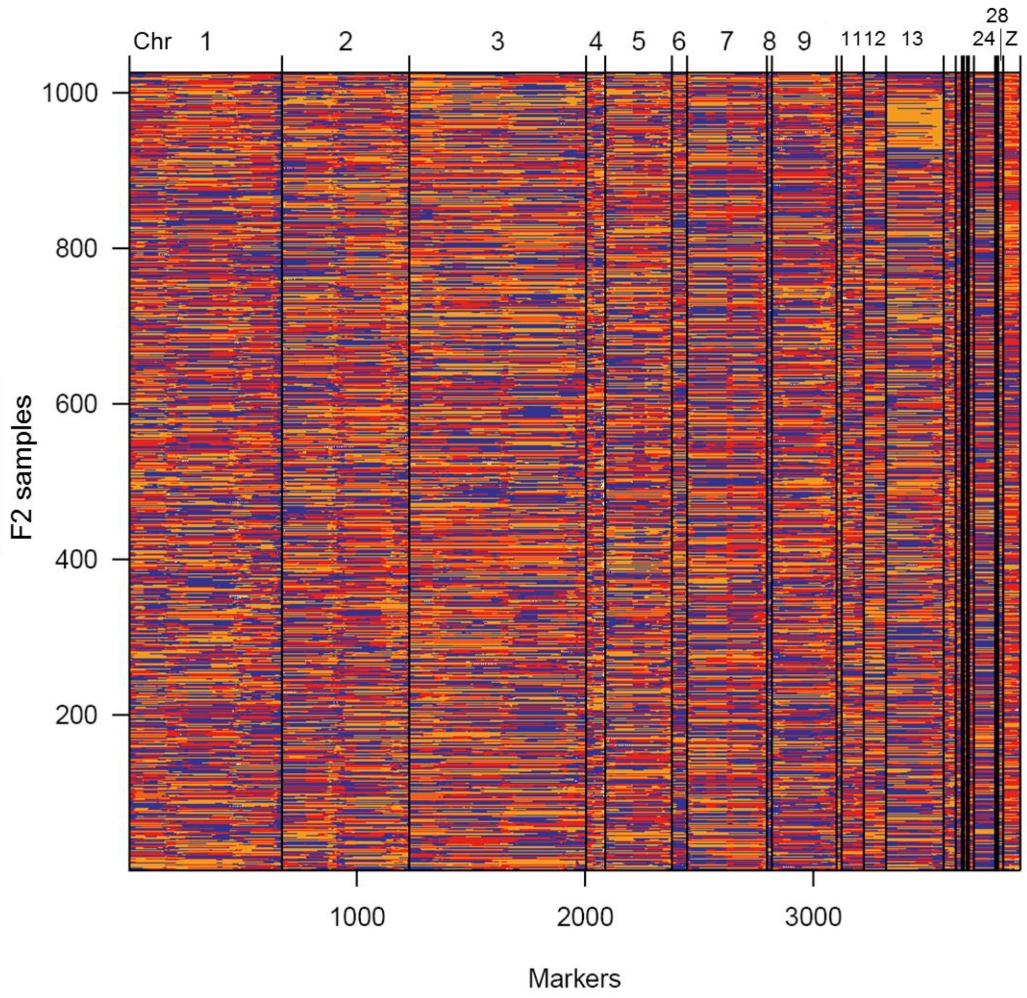

Supplementary Figure 11. The bin map of the 1026-individual F_2 population from the intercross of mallards and Pekin ducks. The red, orange, and blue refer to the Pekin, heterozygotic, and mallard genotypes, respectively. A total of 3,905 bin markers were identified in the whole genome. The width of each column is equal to the genetic distance of the corresponding chromosome.

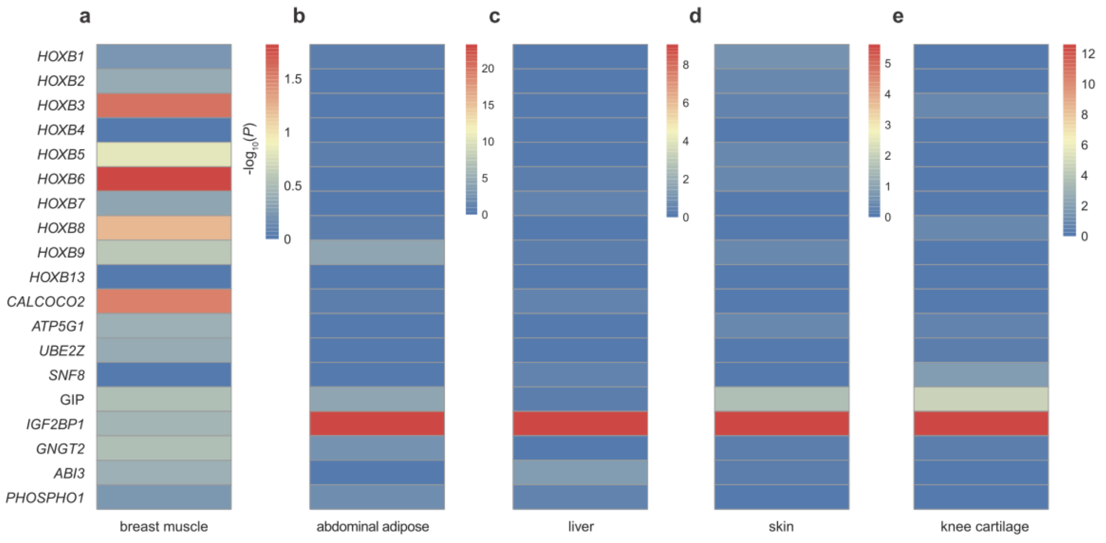

Supplementary Figure 12. Differential gene expression between mallard and Pekin duck at the end of chromosome 28 in multiple tissues. (**a**) Breast muscle, (**b**) abdominal adipose tissue, (**c**) liver, (**d**) skin, and (**e**) knee cartilage. The *P* value was obtained from mallards vs. Pekin ducks using RNA-seq data (**Supplementary Dataset 3**). The -log (*P*) is shown. *P* values obtained from Fisher's exact test using edgeR.

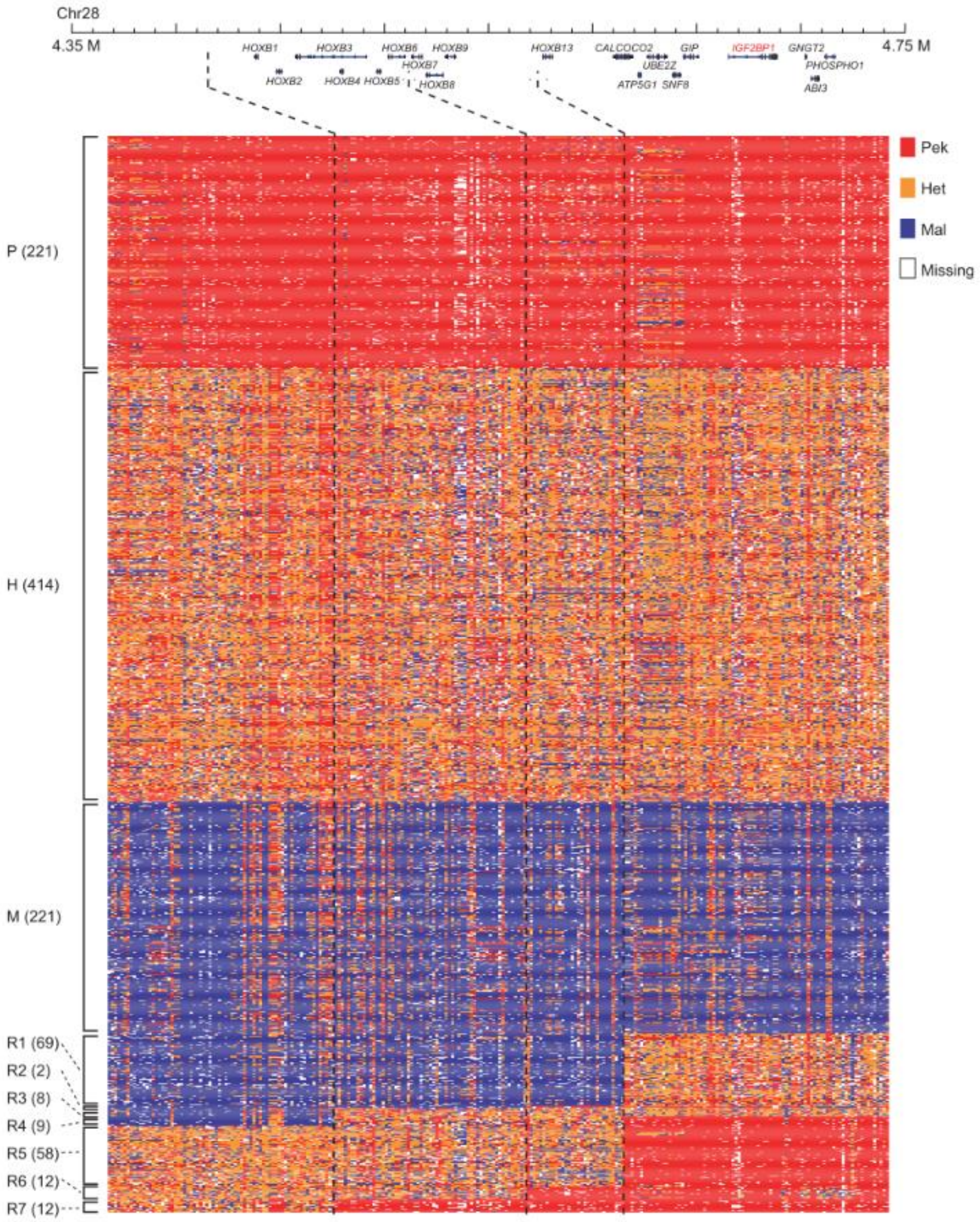

Supplementary Figure 13. Regional recombination map of 1026 F_2 individuals. The top refers to the gene structure of the ends of chromosome 28. The black dashed lines refer to breakpoints 1, 2, and 3, respectively. The red, orange, blue, and white refer to mallard (M), Pekin (P), heterozygotic (H), and missing genotypes, respectively. R1-7 refer to 7 recombinant types, and the abbreviations on the left of the plot refer to different recombinant types. The numbers in brackets refer to the numbers of recombinant individuals.

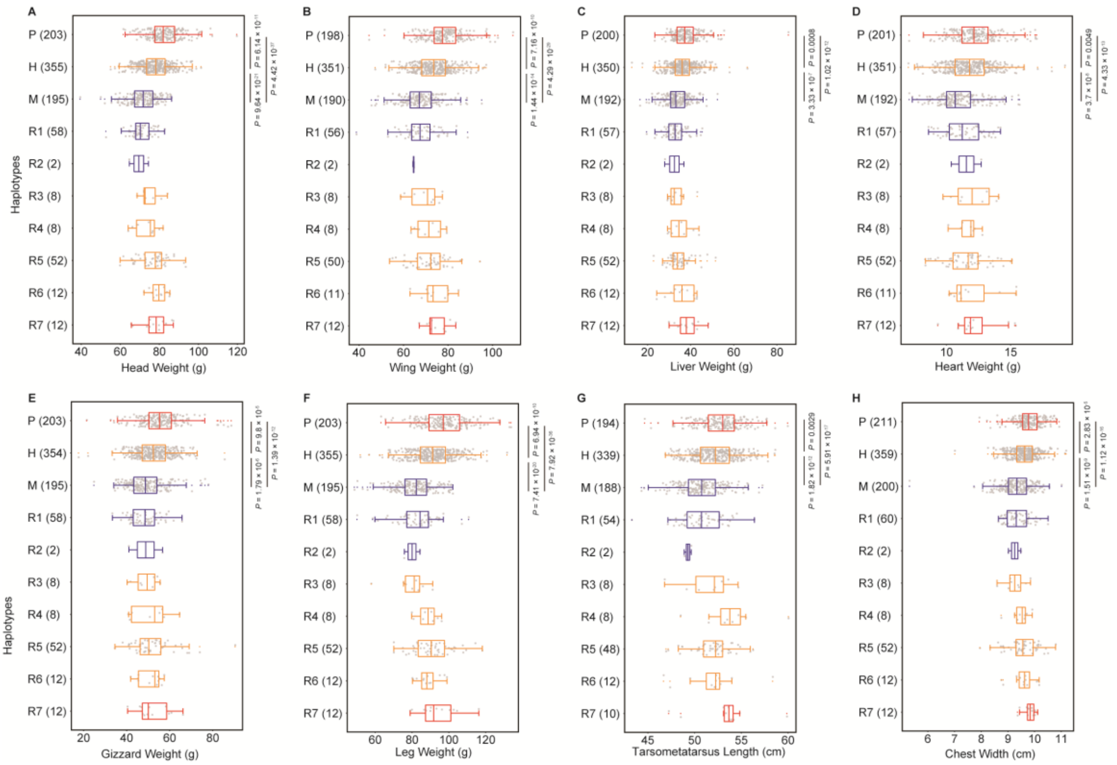

Supplementary Figure 14. Box plots of the phenotype distribution of body size-related traits along the 10 recombination types. (**a**) Head weight, (**b**) wing weight, (**c**) liver weight, (**d**) heart weight, (**e**) gizzard weight, (**f**) leg weight, (**g**) tarsometatarsus length, and (**h**) chest width of 10 recombinant types. The number of individuals with each phenotype is shown in brackets. *P* values are indicated (one-way ANOVA). Box plots denote median (center line), 25–75th percentile (limits), minimum and maximum values without outliers (whiskers), and outliers (red and blue dots).

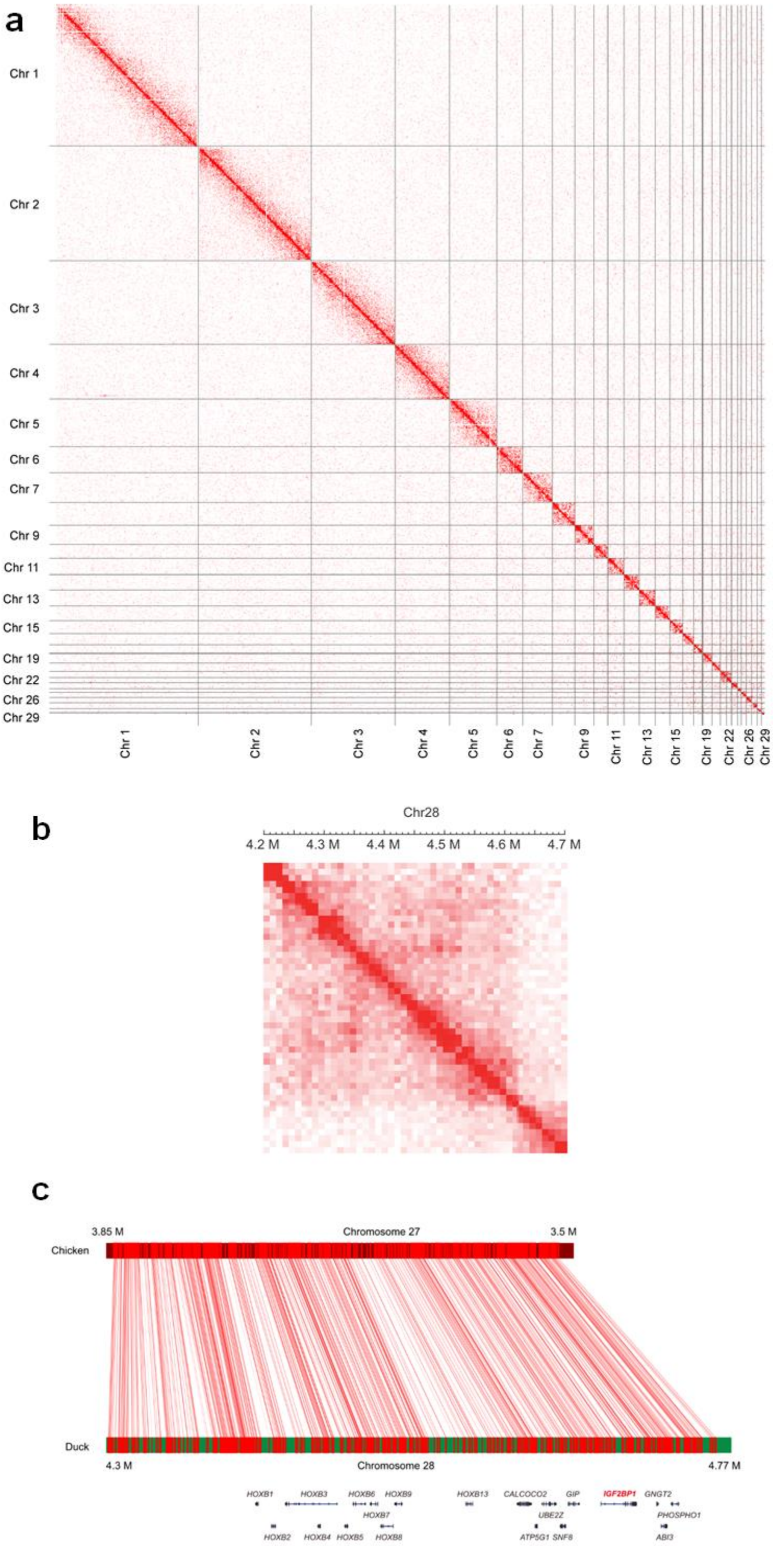

Supplementary Figure 15. Chromosome interaction mapping (Hi-C) and collinearity analysis confirm the scaffold assembly of the end of chromosome 28. (**a**) Whole-genome chromatin interaction based on Hi-C analysis. Strong contacts are shown in red, and weak contacts are shown in white. (**b**) Chromatin interactions at the end of chromosome 28. The heat map shows a normalized contact matrix in 10-kb

bins. (**c**) Collinearity between chicken and duck at the end of chromosome 28.

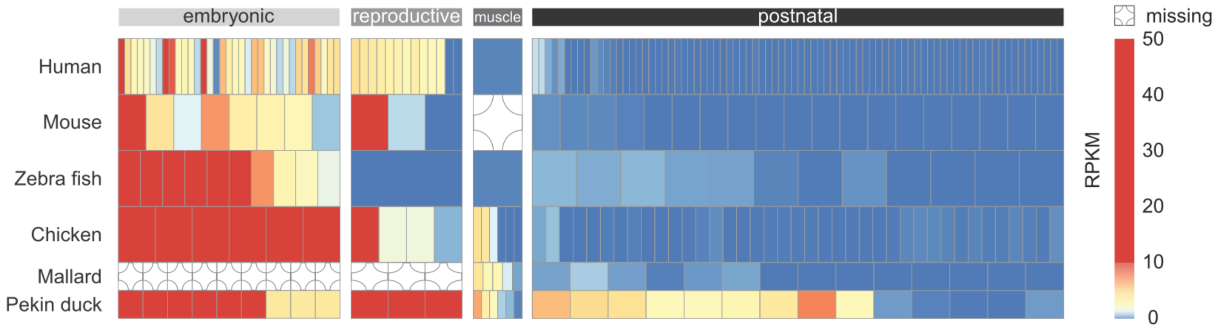

Supplementary Figure 16. The expression pattern of *IGF2BP1* in 5 species. The top bars refer to embryonic-period organs (light gray), reproductive organs (dark gray), muscle, and post-hatching organs (black). Each block of the grid denotes the *IGF2BP1* expression level in a sample. The heat map shows consistent *IGF2BP1* expression after hatching in Pekin ducks. All data except for those from ducks were downloaded from the NCBI SRA dataset (**Supplementary Table 17**).

VI. Supplementary Tables

Supplementary Table 1. Breed information for mallards, indigenous breeds, and Pekin ducks. The characteristics, including appearance, growth performance, egg laying performance, and economic type, are listed as follows. The * indicates that the male ducks have a glossy green head.

Breed	Appearance	Characteristics	Distribution
Pekin duck	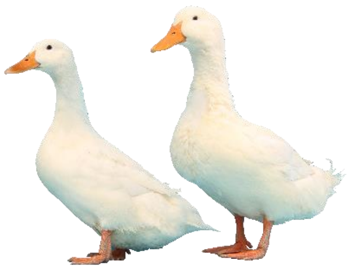	◆ Meat type.<br>◆ A world-famous breed intensively selected for fast growth and efficient accumulation of lean meat.<br>◆ White color. The ducklings are fully covered with golden feathers and become entirely white in adulthood.<br>◆ Large body size. The adult duck weighs approximately 4500 g.<br>Early-maturing type. It starts laying eggs beginning at 165-170 days, with an annual egg production of approximately 220-240.	The origin of Peking ducks is Beijing, China. The breed then spread to other provinces, such as Shandong, Guangdong, and Liaoning, and even to several countries worldwide.

Mallard<br>(wild duck,<br>*Anas platyrhynchos*)*	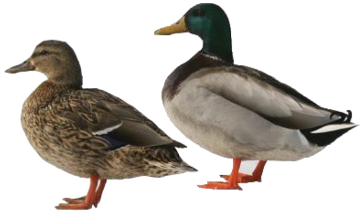	◆ Wild type.<br>◆ Small body size. The adult duck weighs 720-1580 g.<br>◆ The male mallard has a glossy bottle-green head and curled drake feathers, and the other parts are mostly gray. The females are predominantly mottled with dark brown feathers.<br>◆ Flying and migration. It maintains characteristics such as flying and autumn migration.	The mallard breeds throughout the temperate and subtropical Americas, Eurasia, and North Africa and is currently widely distributed across the Northern and Southern Hemispheres.
Shaoxing duck*	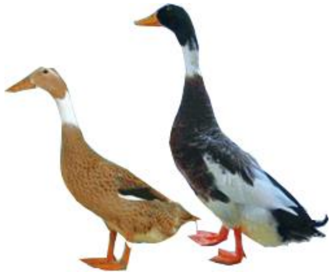	◆ Laying type.<br>◆ The male ducks are dark brown with a glossy bottle-green head. The female ducks have mainly brown-speckled plumage.<br>◆ Small body size. Adult ducks weigh from 1200-1400 g; the male is much heavier than the female.<br>◆ Excellent egg-laying performance. Shaoxing ducks attain maturity early and lay eggs at 104 days of age, with each duck producing an average of 300 eggs per year.	The Shaoxing duck originated in Shaoxing City, Zhejiang Province. At present, the breed is mainly located in Jiangxi, Fujian, Hunan, and Guangdong, among others.

Gaoyou duck*	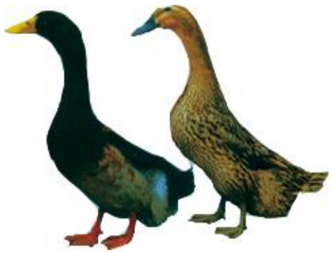	◆ General-purpose type.<br>◆ Large body size. The weight of the adult male duck ranges from 3 to 4 kg, while the female weighs from 2.3 to 3 kg.<br>◆ Fast growth. This breed shows good growth performance and reaches 2.5 kg at only 2 months of age.<br>◆ The female begins laying eggs at 180-210 days, with an average egg production at one year of 170. The egg is approximately 70-80 g and frequently contains double yolks.	This breed is native to Gaoyou City, Jiangsu Province, and spread to other counties along the Beijing-Hangzhou Grand Canal.
Ji’an red duck	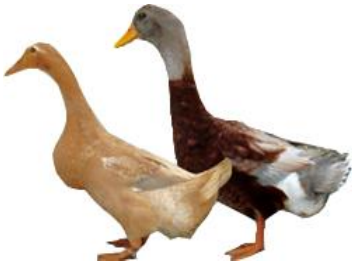	◆ General-purpose type.<br>◆ The body feathers of the male are light red, while those of the female are pale brown.<br>◆ Moderate body size. The Ji’an red duck weighs 1700-1900 g after maturing. It has a high deposition of lean meat and high meat quality.<br>◆ Slow growth type. These ducks grow at a slow rate, similar to mallards.<br>◆ This breed produces an average of 230 eggs annually.	The breed is located in Suichuan and adjacent counties, Jiangxi Province. The distribution areas of Ji'an red duck are fairly small.

Youxian sheldrake*	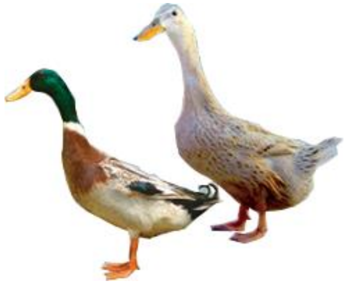	◆ Laying type.<br>◆ Female ducks have mainly light-colored, brown-speckled plumage. The male ducks are similar to the mallard.<br>◆ Small body size. The body is well proportioned, long, and narrow. The weight of the adult duck is 1100-1300 g.<br>◆ Good laying performance. Most ducks become mature and lay eggs at approximately 100 days of age. The number of eggs produced per year is 230-260.	This breed originated in You County, Hunan Province, and then spread to distant areas, such as Guangdong, Hubei, Jiangxi, and Shanghai.
Mawang duck*	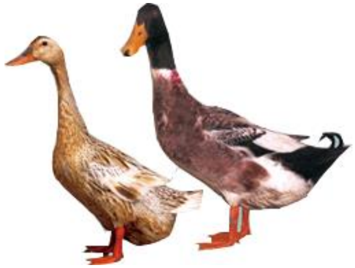	◆ Laying type.<br>◆ Male ducks are dark brown with a glossy bottle-green head. The females have mainly brown-speckled plumage.<br>◆ The body size is relatively larger in egg types. Adult duck weighs 1800-2000 g.<br>◆ Early-maturing type with outstanding laying performance. It lays its first egg at approximately 110 days of age and produces more than 260 eggs each year.	The origin of the Mawang duck is Mawang county, Chongqing. Currently, it remains restricted to Chongqing province.

Longsheng Cui-duck*	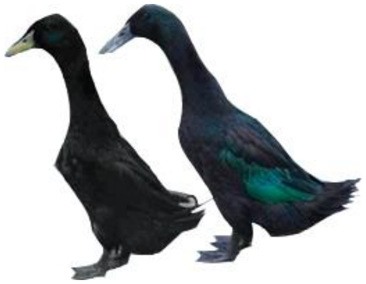	◆ Meat type.<br>◆ Both male and female have glossy black feathers, and the male duck has a malachite green head.<br>◆ The weight of adult ducks is 1800-2000 g, which is larger than most egg types.<br>◆ Starts laying eggs at an average of 170-190 days of age, with a total number of eggs per year ranging from 160-200. The eggshell is blue in general.	This breed originated and is distributed in the multinational autonomous county of Longsheng, Guangxi Province.
Sansui duck*	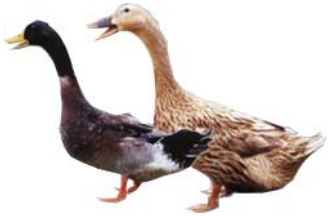	◆ Laying type.<br>◆ The male ducks are dark brown with a bottle-green head. The females have mainly dark brown-speckled plumage.<br>◆ Small size. Small head, tenuous neck, and a body well suited for shipping. Adults are 1400-1700 g.<br>◆ Superior laying performance. The average age of producing the first egg is approximately 130 days, and it produces more than 240 eggs annually.	Sansui ducks are spread throughout several towns in Guizhou.

Jinding duck*	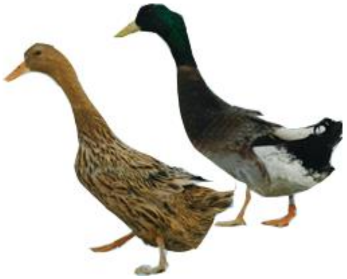	◆ Laying type.<br>◆ The male ducks are brown with a dark green head. The females have mainly black speckled plumage.<br>◆ Adult ducks weigh from 1600-1900 g, and female ducks are larger than males.<br>◆ Early-maturing type. It begins laying eggs at 139 days of age and produces approximately 288 eggs before 500 days.	The center of the distribution area is Jinding village in Longhai county, Fujian province. The breed is also found in Xiamen city.
Liancheng white duck	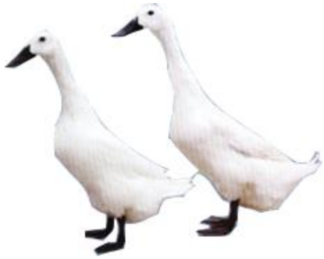	◆ Laying type.<br>◆ The appearance of the male and female is extremely similar. The feathers are almost white, while the beak, shank, and claws are black. Small body size. The body weight of adult ducks is 1300-1500 g.<br>◆ Outstanding egg production. Liancheng white ducks can produce 250-280 eggs per year, and the average weight of their eggs is 70 g.	Liancheng white ducks have inhabited Liancheng, Fujian Province, for more than one hundred years.
Putian black duck	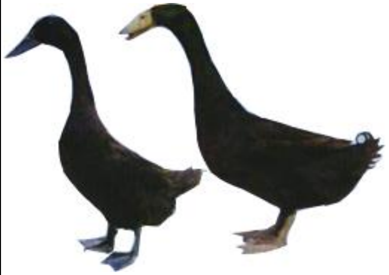	◆ Laying type.<br>◆ It is the only indigenous breed with black plumage on the whole body.<br>◆ The figure is slender and lively. The weight of the adult duck is between 1300 and 1600 g. The eggs of this breed are slightly heavier than those of most other laying breeds.	Putian black ducks are distributed only in Putian County, Fujian Province.

		◆ Most ducks lay eggs from 120 days of age and produce approximately 300 eggs at 500 days. A blue eggshell is one of the hereditary characteristics of this breed.	
Shan sheldrake*	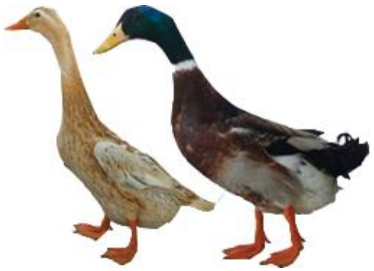	◆ Laying type.<br>◆ The male ducks have a shiny green head and taupe brown feathers from back to waist. Females have mainly brown or light brown speckled plumage.<br>◆ Small body size. Both males and females weigh 1500 g when mature.<br>◆ Excellent egg-laying performance. By 500 days of age, Shan sheldrakes can produce nearly 300 eggs.<br>Fit for grazing in terraced fields because of its small body size and good running and climbing ability.	The center of the geographical range is Longyan City, Fujian Province.
Taiwan sheldrake*	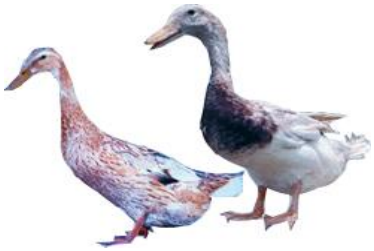	Laying type.<br>The female ducks have light brown feathers covering the whole body. In males, the feathers on the chest are dark gray, and those on other parts are white or gray.<br>The weight of the adult female is 1000-1200 g, and the male weighs 200-300 g more.<br>These ducks produce approximately 250 eggs per year.	Taiwan sheldrakes mainly live in Yilan, Dalin, and Pingdong, Taiwan Province.

Supplementary Table 2. Summary statistics of the duck genome chromosome-level assembly. The scaffolds on the genome assembly BGI_duck_1.0 were anchored to 32 chromosomes, consisting of 29 autosomes along with chromosomes Z, W, and U. Chromosome U comprised scaffolds that were unsuccessfully assembled to other chromosomes. The length and the number of genes for each chromosome are listed in the table.

Chromosome	Length (bp)	Number of genes
1	198,279,714	2,121
2	154,284,905	1,355
3	115,726,694	1,210
4	74,522,697	760
5	63,518,290	950
6	36,432,895	505
7	39,267,675	502
8	31,227,811	518
9	26,143,058	448
10	18,705,356	315
11	21,689,149	423
12	20,948,878	349
13	21,836,115	320
14	19,493,316	344
15	17,611,747	405
16	15,016,072	372
17	387,063	39
18	11,812,398	296
19	12,467,614	308
20	11,802,570	319
21	15,674,435	338
22	7,938,656	247
23	4,481,959	108
24	7,225,183	217
25	7,330,073	159
26	1,284,139	84
27	6,461,911	253
28	4,767,862	196
29	4,453,915	192
Z	74,036,464	708
W	1,096,236	16
U (unplaced)	41,995,137	1,535
Total	1,087,919,987	15,912

Supplementary Table 3. Mapping details of 106 resequencing samples. The mapping rate, insert size, and average depth per sample are provided.

Sample	Breed	Mapping rate	Insert size	Average depth
10	Mallard	0.90	464	11.96
102	Mallard	0.89	501	10.26
16	Mallard	0.89	529	9.97
17	Mallard	0.91	503	10.52
18	Mallard	0.89	501	10.47
21	Mallard	0.88	504	11.23
25	Mallard	0.88	493	9.22
26	Mallard	0.90	466	10.87
28	Mallard	0.89	496	9.67
29	Mallard	0.89	530	9.62
32	Mallard	0.91	497	10.15
33	Mallard	0.89	512	10.48
34	Mallard	0.90	503	11.54
45	Mallard	0.89	515	11.56
46	Mallard	0.89	525	9.45
49	Mallard	0.91	496	9.71
51	Mallard	0.90	500	10.84
52	Mallard	0.88	529	9.18
53	Mallard	0.89	508	9.44
58	Mallard	0.89	497	10.30
59	Mallard	0.89	475	10.32
6	Mallard	0.91	503	11.75
64	Mallard	0.89	501	10.14
65	Mallard	0.90	530	10.37
7	Mallard	0.89	485	10.75
70	Mallard	0.88	509	8.73
71	Mallard	0.89	524	8.18
76	Mallard	0.89	522	9.65
78	Mallard	0.89	499	12.39
80	Mallard	0.90	501	10.69
82	Mallard	0.88	508	10.51
83	Mallard	0.89	522	10.25
84	Mallard	0.89	497	11.30
9	Mallard	0.90	498	11.37
90	Mallard	0.89	524	10.45
95	Mallard	0.89	523	10.33
96	Mallard	0.89	527	9.93

B	Mallard	0.79	285	12.14
C	Mallard	0.89	287	12.89
D	Mallard	0.78	285	12.92
1-2a	Pekin duck	0.90	478	8.92
1-2b	Pekin duck	0.87	463	9.47
1-3a	Pekin duck	0.88	474	11.03
1-3b	Pekin duck	0.89	465	11.93
2-1a	Pekin duck	0.88	477	12.64
2-1b	Pekin duck	0.88	481	11.64
2-2a	Pekin duck	0.88	465	11.78
2-2b	Pekin duck	0.90	475	8.94
2-3a	Pekin duck	0.87	460	10.85
2-3b	Pekin duck	0.87	459	12.02
5-3a	Pekin duck	0.89	468	10.88
5-3b	Pekin duck	0.88	471	9.88
PK-10	Pekin duck	0.86	506	12.19
PK-11	Pekin duck	0.86	474	12.69
PK-12	Pekin duck	0.86	491	11.04
PK-13	Pekin duck	0.88	476	10.96
PK-14	Pekin duck	0.86	507	9.18
PK-2	Pekin duck	0.86	508	9.88
PK-3	Pekin duck	0.86	534	8.07
PK-4	Pekin duck	0.87	511	9.04
PK-5	Pekin duck	0.86	501	11.69
PK-6	Pekin duck	0.86	501	14.10
yz-1a	Pekin duck	0.87	464	10.22
yz-1b	Pekin duck	0.88	466	11.69
yz-2a	Pekin duck	0.88	462	10.98
yz-2b	Pekin duck	0.89	484	11.38
yz-3a	Pekin duck	0.88	468	11.76
yz-3b	Pekin duck	0.89	471	13.50
yz-4a	Pekin duck	0.87	474	11.26
yz-4b	Pekin duck	0.88	458	11.40
GY1	Gaoyou duck	0.81	351	9.40
GY2	Gaoyou duck	0.79	337	8.28
GY-ci3	Gaoyou duck	0.88	308	14.69
GA1	Ji'an red duck	0.84	317	11.29
GA2	Ji'an red duck	0.82	333	7.46
GA-ci2	Ji'an red duck	0.89	314	15.75
JD1	Jinding duck	0.86	477	10.03
JD2	Jinding duck	0.85	489	9.36
JD-ci2	Jinding duck	0.89	333	12.10

LC1	Liancheng white duck	0.82	335	9.21
LC2	Liancheng white duck	0.81	342	7.62
LC-ci2	Liancheng white duck	0.88	325	17.14
LS1	Longsheng jade-green duck	0.82	312	8.23
LS2	Longsheng jade-green duck	0.82	300	9.29
LS-ci2	Longsheng jade-green duck	0.88	340	12.60
MW1	Mawang duck	0.81	342	8.67
MW2	Mawang duck	0.82	326	10.13
MW-ci2	Mawang duck	0.87	337	12.38
PT1	Putian black duck	0.82	310	9.05
PT2	Putian black duck	0.82	309	8.76
PT-ci2	Putian black duck	0.86	343	16.52
SS1	Sansui duck	0.82	347	7.96
SS2	Sansui duck	0.82	334	9.12
SS-ci2	Sansui duck	0.87	324	14.23
SM1	Shan sheldrake	0.83	339	8.64
SM2	Shan sheldrake	0.82	336	10.85
SM-ci2	Shan sheldrake	0.87	321	9.18
SX1	Shaoxing duck	0.83	309	8.82
SX2	Shaoxing duck	0.81	313	8.54
SX-ci2	Shaoxing duck	0.88	343	12.63
TW1	Taiwan duck	0.82	313	9.11
TW2	Taiwan duck	0.82	307	9.54
TW-ci2	Taiwan duck	0.87	349	13.32
YX1	Youxian sheldrake	0.81	340	8.29
YX2	Youxian sheldrake	0.81	342	8.71
YX-ci2	Youxian sheldrake	0.88	325	18.69

Supplementary Table 4. Distributions of SNPs and indels detected by whole-genome resequencing (106 samples). SNPs and indels were identified as splicing, noncoding, exon, UTR (5' UTR or 3' UTR), up-downstream (upstream or downstream of the gene), intergenic, intronic, and others.

Position	Autosome		Sex chromosome	
	SNP count	Indel count	SNP count	Indel count
splicing	919	0	19	2
noncoding	58,801	87	1,407	75
exon	179,250	3,035	3,706	0
UTR	255,872	18,089	3,280	258
up-downstream	2,464,446	161,478	53,119	4,004
intergenic	3,123,735	195,728	164,710	11,686
intronic	6,236,881	422,243	145,599	11,272
others	29,425	3,637	655	93
total	12,349,329	804,297	372,495	27,390

Supplementary Table 5. Comparison of three demographic models using fastsimcoal2 (Mal for mallard, Ind for indigenous breeds, and Pek for Pekin duck). Three null models were designed to infer the process of duck domestication, log-likelihood values, and AIC values obtained for all models. The maximum observed likelihood (MaxObsLhood) corresponds to the likelihood of a perfect fit to the observed data. The maximum estimated likelihood (MaxEstLhood) is the likelihood estimated based on the expected SFS according to the parameters in the optimization. Model 1 provided the largest likelihood value among all three models and thus was chosen as the best fit model.

Alternative models	Model 1: Mal[1] diverged first, then Ind[2], Pek[3]	Model 2: Pek diverged first, then Mal, Ind	Model 3: Ind diverged first, then Mal, Pek
Pattern	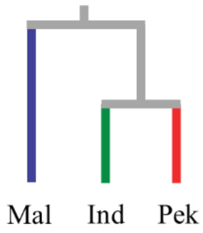	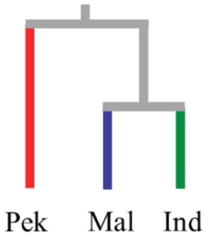	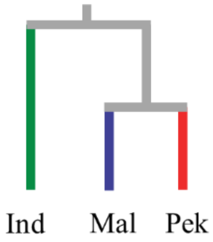
MaxEstLhood	-8502752.56	-9297171.316	-8887060.26
AIC	39156636.59	42815070.16	40926438.95
PS: MaxObsLhood = -7660257.067			

[1] Mallard

[2] Indigenous breeds

[3] Pekin duck

Supplementary Table 6. Parameters and results inferred by ∂a∂i simulations.

Parameters estimated by ∂a∂i are listed. All the parameters are presented with a 95% confidence interval (95% CI), and all the effective population sizes are presented as the number of diploid individuals. The times are estimated with a mutation rate of 1.8 $\times 10^{-9}$ mutations/generation/site and a generation time of 1 year.

**Events**	**Item symbols**	**Values**	**Confidence interval (95%)**	**Units**
Time between first split and second split	Ta	764.06	(740.74,787.39)	year
Time between second split and present	Tb	918.59	(888.62,948.56)	year
Population size of non-Mal at T2	nu_dom	10109.14	(9847.54,10370.75)	individual
Population size of Mal at present	nu1	18819.59	(18723.49,18915.68)	Individual
Population size of Ind at T2	nu2_0	3401.78	(3391.87,3411.69)	individual
Population size of Ind at present	nu2	23905.10	(23876.51,23933.70)	individual
Population size of Pek at T2	nu3_0	2534.90	(2266.48,2803.32)	individual
Population size of Pek at present	nu3	3577.86	(3510.67,3645.05)	individual
Migration from Pek to Ind between T2 and present	m1	5.47E-08	nan	individual/year
Migration from Ind to Pek between T2 and present	m2	1.59E-06	nan	individual/year

Supplementary Table 7. D-statistics of D (Muscovy duck (W), Pekin duck (X), GY (Y), Z). A positive D statistic value indicates that the X and Z populations share more derived alleles with each other than do X and Y, while a negative value indicates that the X and Y populations share more derived alleles than do X and Z.

W	X	Y	Z	D value	Z score
Muscovy duck	Pekin duck	GY	SX(Z)	-0.026	-7.737
Muscovy duck	Pekin duck	GY	JD(Z)	-0.0366	-9.459
Muscovy duck	Pekin duck	GY	SS(Z)	-0.0266	-6.698
Muscovy duck	Pekin duck	GY	SM(Z)	-0.0495	-12.385
Muscovy duck	Pekin duck	GY	YX(Z)	-0.0451	-11.525
Muscovy duck	Pekin duck	GY	MW(Z)	-0.0444	-10.776
Muscovy duck	Pekin duck	GY	TW(Z)	-0.0418	-10.19
Muscovy duck	Pekin duck	GY	LC(Z)	-0.048	-11.215
Muscovy duck	Pekin duck	GY	PT(Z)	-0.0396	-10.018
Muscovy duck	Pekin duck	GY	JA(Z)	-0.0235	-5.252
Muscovy duck	Pekin duck	GY	LS(Z)	-0.0144	-3.365

Supplementary Table 8. The mean and threshold of the F_{ST} and π ln ratio.

**Population**	**Mean weighted F_{ST}**	**Mean π ln ratio**	**Threshold of F_{ST} (P < 0.005)**	**Threshold of π ln ratio (P < 0.005)**	**Fifth maximum of permutation value for F_{ST} [4]**	**Top 10 maximum permutation values for F_{ST}**
Mal[1] vs. Ind[2]	0.070332	0.05557588	0.207019	0.695007883	0.158785	0.17619, 0.173252, 0.166365, 0.164028, 0.158785, 0.157641, 0.151817, 0.150805, 0.138721, 0.13601
Ind vs. Pek[3]	0.10357	0.190408321	0.30449	1.229149769	0.193225	0.222753, 0.220095, 0.219102, 0.204254, 0.193225, 0.192792, 0.191981, 0.190458, 0.19019, 0.184626

[1]Mallard

[2]Indigenous breeds

[3]Pekin duck

[4]We performed 100 permutations and sorted the 100 maximum values from largest to smallest. Finally, we took the fifth value as the final permutation threshold.

Supplementary Table 9. Genomic regions identified as CDRs between mallard and indigenous ducks. Max F_{ST} refers to the maximal windowed F_{ST} value of the CDRs. The ln ratio ($\theta_{\pi,\ mallards} / \theta_{\pi,\ indigenous\ breeds}$) refers to the maximal π ln ratio value of the CDRs. Fixed SNPs represent sites with standing variations in mallard but that are nearly fixed in indigenous breeds. Candidate genes are shown for genes with enrichment by KEGG residing in these regions.

No.	Chrom	Start (bp)	End (bp)	Max F_{ST}	ln ratio ($\theta_{\pi,\ mallards} / \theta_{\pi,\ indigenous\ breeds}$)	Fixed SNP number	Total SNP number	Fixed SNP number / total SNP number	Candidate gene
1	1	21160001	21720000	0.300475	2.287011929	10	1602	0.006242197	*GRM8*
2	1	29010001	29120000	0.25488	1.002955037	1	317	0.003154574	
3	1	29430001	29530000	0.301264	2.342987458	30	784	0.038265306	*NRCAM, CNTN1*
4	1	65140001	65210000	0.224148	1.367561573	0	181	0	*MGST1*
5	1	77590001	77870000	0.250789	1.8032917	8	1335	0.005992509	*YBX3, GABARAPL1, LOC101801364, LOC101801176, LOC101801552, A2M, M6PR*
6	1	84450001	84540000	0.258221	0.727323983	2	849	0.002355713	
7	1	91410001	91450000	0.209115	0.857587816	2	240	0.008333333	
8	1	95600001	96670000	0.377001	2.965640467	26	5054	0.00514444	*GBE1*
9	1	97640001	97880000	0.33912	0.78793248	6	1212	0.004950495	*ROBO1*
10	1	100120001	100550000	0.462398	1.471619097	15	2502	0.005995204	
11	1	101270001	101330000	0.253572	1.209130058	17	273	0.062271062	
12	1	101660001	104060000	0.386589	2.35319483	90	9399	0.009575487	*NCAM2, JAM2, ATP5J, APP*
13	1	104400001	105210000	0.32067	0.912427126	3	3226	0.000929944	
14	1	105730001	105870000	0.254241	0.829030846	2	1380	0.001449275	*GRIK1*

15	1	107200001	107480000	0.373234	1.012103689	4	1694	0.002361275	*ATP5O, MRPS6, KCNE2, KCNE1, RCAN1*
16	1	107690001	107820000	0.383275	1.109870656	1	922	0.001084599	*RUNX1*
17	1	108290001	108450000	0.340768	1.152278223	1	1003	0.000997009	*LOC101794661, CLDN14, HLCS*
18	1	173940001	174040000	0.230289	0.881614064	6	1360	0.004411765	*LOC101798073*
19	1	176300001	176460000	0.314384	0.824333815	0	910	0	
20	1	180830001	181210000	0.266433	0.758645916	1	1531	0.000653168	*FGF9*
21	1	182510001	182560000	0.210161	0.714352072	0	334	0	*RDX*
22	1	185960001	186080000	0.242698	1.266678126	0	417	0	*TRPC6, PGR*
23	2	1180001	1230000	0.229861	1.481284604	3	538	0.005576208	*PTH1R, MYL3*
24	2	3890001	3990000	0.280068	1.12223539	6	1068	0.005617978	*AQP1*
25	2	15320001	15370000	0.225249	1.944845293	0	273	0	
26	2	27210001	27400000	0.243441	1.153796849	1	610	0.001639344	
27	2	29790001	30160000	0.298635	0.793509145	3	1153	0.002601908	
28	2	38790001	39190000	0.331155	0.775536757	1	2775	0.00036036	*LOC106019265, LOC101794899*
29	2	57280001	57360000	0.26836	0.987128411	0	164	0	*GLI3*
30	2	64990001	65060000	0.242242	1.172709189	0	73	0	
31	2	65660001	65820000	0.25472	1.072399354	1	785	0.001273885	*PDCD6IP*
32	2	74130001	74440000	0.319112	1.113615995	12	1456	0.008241758	
33	2	75490001	75920000	0.334015	1.227782983	6	1263	0.004750594	
34	2	76490001	76850000	0.385543	2.589916639	139	2807	0.049519059	*DROSHA, MC4R*
35	2	79830001	79980000	0.320143	0.977825061	1	846	0.001182033	
36	2	85760001	86250000	0.3072	1.290045371	1	1652	0.000605327	
37	2	93480001	93790000	0.276326	0.751657696	3	3840	0.00078125	*ALG2, PARD6G*
38	2	133300001	133410000	0.310029	0.711558065	3	1164	0.00257732	
39	2	144030001	144360000	0.289219	1.840132498	0	1988	0	
40	2	148870001	148940000	0.266552	1.058154186	4	486	0.008230453	

41	2	151750001	151920000	0.294956	1.800981931	30	2234	0.013428827	
42	3	3430001	3490000	0.24395	1.890476439	3	306	0.009803922	
43	3	17640001	17880000	0.217564	1.142429687	0	1643	0	*KCNK2*
44	3	24130001	24180000	0.229913	1.457882273	0	174	0	*GNG4*
45	3	28650001	28700000	0.224207	1.447340496	0	321	0	*SRD5A2*
46	3	36650001	36910000	0.352775	1.260366013	20	1211	0.016515277	*PRKCE, LOC106015636*
47	3	37600001	37960000	0.278426	2.220724514	3	1967	0.001525165	*ABCG8, ABCG5, DYNC2LI1*
48	3	44050001	44960000	0.336715	1.018991101	15	4782	0.003136763	
49	3	46340001	46860000	0.420123	0.915422427	31	3574	0.008673755	*AGPAT4, MAP3K4*
50	3	47810001	47880000	0.236483	0.896155189	6	568	0.01056338	
51	3	49940001	50040000	0.272735	0.920615576	5	1159	0.004314064	*UST, TAB2, LATS1, NUP43*
52	3	50260001	50310000	0.223229	1.20064243	3	539	0.005565863	*IYD*
53	3	50610001	50910000	0.254761	1.183149957	8	2568	0.003115265	*MTHFD1L, ESR1*
54	3	52550001	53140000	0.341495	0.972691684	17	4058	0.004189256	*CLDN20, NOX3*
55	3	53640001	53780000	0.246899	0.705601775	18	1723	0.010446895	*SYNJ2*
56	3	59190001	59620000	0.351941	1.416277161	17	3430	0.004956268	*RPS12, LOC101798418, LOC101798228, LOC101798651, TAAR2, LOC101789807, STX7, CTGF*
57	3	59860001	60050000	0.290595	1.50447226	28	1759	0.015918135	*EPB41L2*
58	3	60680001	60760000	0.267663	0.945225413	2	564	0.003546099	*LAMA2*
59	3	71390001	71910000	0.349327	2.486955628	115	5424	0.021202065	*PREP*
60	3	75070001	75630000	0.314616	0.800257351	6	2955	0.002030457	
61	3	78810001	78880000	0.327157	1.449397986	39	876	0.044520548	*MDN1*
62	3	97960001	98000000	0.209487	0.900356035	0	77	0	
63	4	20550001	21320000	0.349096	1.435607666	2	2289	0.000873744	
64	4	22520001	22630000	0.232672	0.716191485	0	367	0	*VEGFC, SPCS3*
65	4	23710001	23910000	0.320957	0.790203138	0	497	0	*GALNT7*

66	4	28840001	29280000	0.406315	1.857975106	37	2150	0.017209302	*RXFP1, GRIA2, GLRB, PDGFC*
67	4	35720001	35790000	0.279277	1.063498389	7	765	0.009150327	*MAML3, MGST2, SETD7*
68	4	36430001	36510000	0.290741	1.106181498	0	273	0	
69	4	37840001	38050000	0.287564	0.819901129	1	850	0.001176471	
70	4	38430001	39680000	0.378135	1.309076511	4	3628	0.001102536	
71	4	47760001	47820000	0.219028	0.70956698	1	729	0.001371742	*AFF1, PTPN13, MAPK10*
72	4	48790001	48840000	0.219433	0.854476719	2	470	0.004255319	*TEC, TXK, CNGA1*
73	4	52380001	52540000	0.267792	0.798766781	4	1682	0.002378121	*PGM2*
74	4	57580001	57740000	0.269924	0.734723607	1	876	0.001141553	
75	4	67280001	67320000	0.212101	0.983991625	2	424	0.004716981	
76	4	68650001	68840000	0.293259	1.419636203	4	1834	0.002181025	
77	4	69670001	69720000	0.210398	1.134718526	2	566	0.003533569	*LOC106018247, EIF2AK3, RPIA, SUCLG1*
78	4	70340001	70450000	0.218331	1.134576227	4	725	0.005517241	
79	5	20670001	20890000	0.306736	1.341615919	0	491	0	*PRKD1*
80	5	21290001	21360000	0.269322	1.585822896	0	84	0	*FOXG1*
81	5	21570001	21960000	0.331649	1.409379436	1	581	0.00172117	
82	5	42780001	42820000	0.211265	1.965550334	0	125	0	*TSHR, GTF2A1, SEL1L*
83	5	46260001	46450000	0.24493	0.708881138	15	1328	0.011295181	*PSMC1, CALM1*
84	5	51450001	51550000	0.321327	0.96689739	0	364	0	*WARS*
85	5	51940001	52000000	0.220832	1.477604397	0	99	0	
86	5	53770001	54010000	0.283481	1.336010275	26	1939	0.013408974	
87	6	11660001	12130000	0.332409	1.242646759	14	2621	0.005341473	*PPIF*
88	6	12830001	12950000	0.300081	0.905558822	1	199	0.005025126	*RPS24, POLR3A, KCNMA1*
89	6	16270001	16700000	0.486211	1.6355074	68	1783	0.03813797	*SORBS1, PIK3AP1, LOC101794817, GBF1, NFKB2, PSD*
90	6	17210001	17280000	0.235511	0.812054395	0	185	0	*NDUFB8, SEC31B, WNT8B, SCD, PKD2L1, LOC101797254, LOC101797666, ALOX5*

91	6	17800001	17980000	0.270306	1.216913705	0	244	0	*MAPK8*
92	6	18560001	18600000	0.211983	0.798697762	0	165	0	*LOC106017909*
93	6	25480001	25520000	0.208257	1.00078721	0	304	0	
94	6	26450001	26820000	0.214645	1.228985409	3	3154	0.000951173	*GPAM, ACSL5, VTI1A*
95	7	14550001	14830000	0.248742	0.93632631	2	923	0.002166847	*ITGA4, UBE2E3*
96	7	36430001	36550000	0.328254	1.521247241	27	1832	0.014737991	*ACVR2A*
97	8	13730001	14590000	0.405258	4.87265919	178	1183	0.15046492	*PLA2G4A, PTGS2, TPR, LOC101799213, RGL1*
98	9	70001	990000	0.359625	1.46581218	2	1837	0.001088732	*PIGZ, NCBP2, LOC101797837, AGTR1, PLOD2*
99	9	10030001	10220000	0.210822	0.825825327	2	1948	0.001026694	*EHHADH, MAP3K13, ANAPC13, EPHB1*
100	9	19920001	20000000	0.228604	1.768853745	11	646	0.017027864	
101	9	21430001	21470000	0.214976	1.349604385	12	546	0.021978022	*PRKCI, SEC62*
102	10	380001	420000	0.207631	1.196755206	3	359	0.008356546	*LOC101802562, HEPH, LOC101803913, LOC101804094, EDA2R*
103	10	5180001	5240000	0.219318	1.190149263	2	143	0.013986014	
104	11	50001	550000	0.275215	1.530641295	0	1100	0	*LOC101791068*
105	12	860001	1020000	0.292577	2.422784847	12	216	0.055555556	
106	12	1320001	1510000	0.270972	1.06406578	3	605	0.004958678	
107	12	11690001	12290000	0.402089	1.121888201	10	2162	0.004625347	*CEBPG, FAAP24, SLC7A9, POLR2C, CNGB1*
108	12	16020001	16540000	0.345822	3.28845404	44	2400	0.018333333	*GCSH, BCO1, PLCG2, HSD17B2, LOC101791461, LOC101791850, LOC101791654, MPHOSPH6*
109	12	17370001	17430000	0.242113	0.92436562	7	659	0.010622155	
110	13	17960001	18000000	0.208963	1.020537905	2	411	0.00486618	*GXYLT2*
111	14	3590001	4220000	0.367649	1.135392593	3	4155	0.000722022	
112	14	4730001	4780000	0.24086	0.950673149	1	333	0.003003003	*SLIT3*
113	16	30001	1180000	0.376409	2.037961879	59	1849	0.03190914	*AACS*

114	16	1610001	1780000	0.275028	1.078038921	4	898	0.004454343	*FZD10, PIWIL1, STX2, RAN*
115	16	6720001	7010000	0.275757	1.183966241	12	1528	0.007853403	*GNAZ, DEPDC5, PISD, LIMK2, RNF185, HNF1A*
116	16	11110001	11170000	0.251792	0.736467127	10	583	0.017152659	*NCOR2*
117	20	2770001	3130000	0.293098	1.420115518	19	4258	0.004462189	*NCF1, GTF2I, GTF2IRD1*
118	21	1	230000	0.322088	1.206029901	1	423	0.002364066	*ITCH, SRSF6, SNAI1*
119	21	9320001	9420000	0.217879	0.84559833	0	247	0	*RAB22A*
120	21	15490001	15550000	0.267832	0.715602102	2	243	0.008230453	*ELMO2*
121	22	5330001	5370000	0.214979	1.218045326	1	245	0.004081633	
122	25	4440001	4500000	0.244449	0.698238048	2	576	0.003472222	*SC5D*
123	28	4400001	4710000	0.399912	0.727856753	2	562	0.003558719	*ATP5G1, UBE2Z, SNF8, GIP, IGF2BP1, GNGT2, PHOSPHO1*

Supplementary Table 10. Genomic regions identified as CDRs between indigenous ducks and Pekin ducks. Max F_{ST} refers to the maximal windowed F_{ST} value of the CDRs. The ln ratio ($\theta_{\pi,\ \text{indigenous breeds}}$ / $\theta_{\pi,\ \text{Pekin ducks}}$) refers to the maximal π ln ratio value of the CDRs. Candidate genes are shown for genes with enrichment by KEGG residing in these regions.

No.	Chrom	Start (bp)	End (bp)	Max F_{ST}	ln ratio ($\theta_{\pi,\ \text{indigenous breeds}}$ / $\theta_{\pi,\ \text{Pekin ducks}}$)	Candidate gene
1	1	18920001	19010000	0.361567	1.233472387	*ALG12*
2	1	68560001	68980000	0.637692	1.603418462	*LOC101792090, CDKN1B, LOC101796138, DUSP16, LRP6*
3	1	69190001	69620000	0.544855	1.491970601	*ETV6, PPARA*
4	1	69960001	71880000	0.523552	1.273352912	*WNT7B, SMC1B, UPK3A, NUP50, PRR5, PARVG, PARVB, LOC101802708*
5	1	74060001	74380000	0.470318	1.351796583	*KCNJ8, ABCC9, CMAS, ST8SIA1*
6	1	77590001	77870000	0.482783	5.876693622	*YBX3, GABARAPL1, LOC101801364, LOC101801176, LOC101801552, A2M, M6PR*
7	1	83240001	83290000	0.33493	1.481687247	
8	1	95600001	96670000	0.416611	1.883669684	*GBE1*
9	1	128690001	129740000	0.335822	6.11473509	*NLGN4X*
10	1	157100001	157320000	0.468125	1.511182467	*CLN5*
11	2	2370001	2430000	0.326999	1.402089726	*WNT3A, ARF1, GUK1*
12	2	11690001	11730000	0.305341	1.737636352	
13	2	33050001	33210000	0.404322	2.028354754	*NUPL2, TRA2A*
14	2	41320001	41370000	0.326016	1.914755892	

15	2	46970001	47070000	0.386725	1.943874948	*NFATC1*
16	2	81800001	81900000	0.503906	2.023160257	*MARCH6, LOC101802880*
17	2	92400001	92510000	0.408456	2.570184745	*LOC101801689*
18	2	93480001	93790000	0.47566	1.827573275	*ALG2, PARD6G*
19	2	100370001	100870000	0.458464	2.150412759	*MC5R, MC2R, RNMT*
20	3	4770001	4880000	0.487099	1.27798491	*LOC101803492, PEX13, REL, PAPOLG*
21	3	19810001	20090000	0.383255	1.354485087	*TGFB2, LOC101794148*
22	3	37600001	37960000	0.315793	4.548968872	*ABCG8, ABCG5, DYNC2LI1*
23	3	73590001	73800000	0.359518	1.548254983	*GRIK2*
24	3	106100001	106380000	0.353137	2.633629892	*LOC101802417*
25	3	108100001	108520000	0.365086	1.24096672	
26	3	109470001	109530000	0.342116	1.280826449	
27	4	32980001	33120000	0.367264	1.867787564	*NR3C2, ARHGAP10, EDNRA*
28	4	33830001	33960000	0.594017	6.969642157	*LSM6, SMAD1*
29	4	34690001	34970000	0.397483	2.224201785	*GAB1, INPP4B, LOC101803085*
30	4	41290001	41360000	0.331946	1.700529314	
31	4	45070001	45410000	0.489725	5.491313963	*PPAT, PAICS*
32	4	45660001	46970000	0.610509	4.904385109	*NPFFR2, RASSF6, LOC101803817, LOC106014932, EREG, AREG, BMP2K*
33	5	14580001	14670000	0.324769	2.329422673	*RAG2*
34	5	22590001	22680000	0.368555	1.507356395	
35	5	33250001	33570000	0.432651	1.23845586	*CHST14, IVD, PLCB2, PAK6, BUB1B, DRD4, TALDO1*
36	5	35130001	35200000	0.361848	4.875681191	*LOC101799861, CARS, KCNQ1*
37	5	35420001	35590000	0.422338	2.162560188	*KCNQ1, TRPM5, CD81*
38	5	36010001	36050000	0.332335	3.550936836	*MRPL23, LSP1, SYT8*
39	5	40000001	40230000	0.330913	1.42986805	*MLH3, ACYP1, FOS, TGFB3*
40	5	53770001	54010000	0.341983	1.512254469	
41	5	56660001	56720000	0.354086	2.340000305	*PPP2R5E*

42	6	3910001	4130000	0.436854	1.247288429	*HERC4, SIRT1, CTNNA3*
43	6	8600001	8650000	0.321927	1.756462849	
44	6	26450001	26820000	0.528304	5.716069192	*GPAM, ACSL5, VTI1A*
45	7	12420001	12700000	0.452066	1.95359748	*NDUFS1*
46	7	17740001	17890000	0.487072	1.895532201	*LOC101803838, RAPGEF4*
47	7	25370001	25430000	0.328916	1.435270814	*GLI2*
48	7	26580001	26910000	0.599932	1.589790976	*ADCY5, HACD2, MYLK*
49	8	12670001	12730000	0.319031	3.693957955	*LAMC1, LAMC2, NMNAT2, SMG7, NCF2, ARPC5, LOC101800410*
50	9	4630001	4700000	0.395455	1.552037041	*AP1S3*
51	9	8620001	8800000	0.387609	3.534566769	*HS6ST1, UGGT1*
52	9	14890001	15560000	0.421111	2.735939781	*TP63, LOC101804768, PDCD1, PCYT1A, TFRC*
53	10	8320001	8400000	0.387225	1.628143784	*LOC101801033, GLA, RAB9B, LOC101798161, NUP62CL, CLDN2, CNGA2*
54	10	8680001	8720000	0.325989	1.754170786	*GABRA3, LOC101802647*
55	11	11270001	11530000	0.372984	2.186752202	*HDC, GATM, PDE8A, HOMER2*
56	13	10150001	10390000	0.469527	1.358363774	*RUVBL1, SEC61A1, MGLL, MCM2*
57	13	16760001	16850000	0.611512	2.511554634	*MITF*
58	13	20030001	20120000	0.458776	1.305216577	*LOC101794070, SUMF1, ITPR1, BHLHE40*
59	15	4350001	4440000	0.394891	1.956692225	*CARD11*
60	16	4720001	4900000	0.475943	1.590034963	*SEPT5, LOC101803251, CLDN5, CDC45, PPIL2*
61	16	8980001	9040000	0.352295	1.49816279	*LOC101792038*
62	21	8640001	8830000	0.380329	1.274196539	*PPP1R3D, ATP5E, TUBB1, CTSZ, GNAS*
63	26	780001	840000	0.366709	1.402771331	*SETDB1, CERS2, PIP5K1A, PSMD4, PI4KB, RFX5, SLAMF1, NCSTN*
64	28	4400001	4710000	0.516371	4.190865095	*ATP5G1, UBE2Z, SNF8, GIP, IGF2BP1, GNGT2, PHOSPHO1*

Supplementary Table 11. The CDRs that undergo continuous selection with the same trend in frequency change in the domestication (dom) and improvement (imp) stages. Max F_{ST} refers to the maximal windowed F_{ST} value of the CDRs. The ln ratio refers to the maximal π ln ratio value of the CDRs. Candidate genes are shown for genes with enrichment by KEGG residing in these regions.

No.	Chrom	Start (bp)	End (bp)	Max F_{ST} in dom	Max F_{ST} in imp	ln ratio ($\theta_{\pi,\ mallards}$ / $\theta_{\pi,\ indigenous\ breeds}$)	ln ratio ($\theta_{\pi,\ indigenous\ breeds}$ / $\theta_{\pi,\ Pekin\ ducks}$)	Candidate gene
1	1	77590001	77870000	0.250789	0.482783	1.8032917	5.876693622	*YBX3, GABARAPL1, LOC101801364, LOC101801176, LOC101801552, A2M, M6PR*
2	1	95600001	96670000	0.377001	0.416611	2.965640467	1.883669684	*GBE1*
3	2	93480001	93790000	0.276326	0.47566	0.751657696	1.827573275	*ALG2, PARD6G*
4	3	37600001	37960000	0.278426	0.315793	2.220724514	4.548968872	*ABCG8, ABCG5, DYNC2LI1*
5	5	53770001	54010000	0.283481	0.341983	1.336010275	1.512254469	
6	6	26450001	26820000	0.214645	0.528304	1.228985409	5.716069192	*GPAM, ACSL5, VTI1A*
7	28	4400001	4710000	0.399912	0.516371	0.727856753	4.190865095	*ATP5G1, UBE2Z, SNF8, GIP, IGF2BP1, GNGT2, PHOSPHO1*

Supplementary Table 12. Fixed SNPs (F_{ST} = 1) along the duck genome.

Chrom	Position
chr13	16781586, 16782638, 16785084, 16790722, 16792606, 16793796, 16795208, 16803735, 16804693, 16805433, 16808318, 16808587, 16808759, 16808761, 16808919, 16808923, 16808987, 16809089, 16809275, 16809300, 16809688
chr28	4414946, 4418769, 4419705, 4420098, 4420841, 4422027, 4423923, 4635455, 4642736, 4653878, 4654638, 4668615, 4672142, 4679684, 4685145, 4693395, 4697808, 4698863

Supplementary Table 13. Genotype frequency for variants with F_{ST} = 1 along the duck genome in additional samples.

Chrom	Position	Mal[1] allele	Pek[2] allele	27 mallards[3]		60 mallards pooled[4]	
				Frq_{Mal}[5]	Frq_{Pek}[6]	Frq_{Mal}	Frq_{Pek}
13	16781586	C	T	0.9259	0.0741	1	0
13	16782638	T	G	1	0	1	0
13	16785084	C	T	1	0	1	0
13	16790722	G	A	1	0	1	0
13	16792606	T	G	0.9815	0.0185	1	0
13	16793796	G	A	0.9815	0.0185	1	0
13	16795208	C	T	0.9815	0.0185	1	0
13	16803735	G	C	1	0	1	0
13	16804693	G	A	1	0	1	0
13	16805433	C	T	1	0	1	0
13	16808318	G	A	1	0	0.9722	0.0278
13	16808587	A	C	1	0	0.5422	0.4578
13	16808759	C	A	1	0	0.92	0.08
13	16808761	C	T	0.9815	0.0185	1	0
13	16808919	A	G	0.9815	0.0185	0.5588	0.4412
13	16808923	A	G	0.9815	0.0185	1	0
13	16808987	C	A	0.963	0.037	0.6364	0.3636
13	16809089	C	T	0.9815	0.0185	0.9516	0.0484
13	16809275	C	T	1	0	0.9875	0.0125
13	16809300	A	T	1	0	1	0
13	16809688	C	T	0.9815	0.0185	0.9429	0.0571
28	4414946	A	C	1	0	1	0
28	4418769	G	A	1	0	1	0
28	4419705	G	A	1	0	1	0
28	4420098	C	A	1	0	1	0
28	4420841	G	A	1	0	1	0
28	4422027	T	C	1	0	1	0
28	4423923	C	T	0.8704	0.1296	1	0
28	4635455	C	T	0.9815	0.0185	1	0
28	4642736	C	G	0.9259	0.0741	1	0
28	4653878	G	A	0.9444	0.0556	1	0
28	4654638	C	T	0.9815	0.0185	1	0
28	4668615	G	T	0.9815	0.0185	1	0
28	4672142	A	G	1	0	1	0
28	4679684	C	T	1	0	1	0
28	4685145	C	T	1	0	1	0
28	4693395	C	T	0.98	0.02	1	0
28	4697808	T	C	1	0	1	0
28	4698863	C	T	1	0	0.975	0.025

[1]Mallard

[2]Pekin duck

[3]The resequencing data of 27 mallards (SRR3471580 - 606) were downloaded from the National Center for Biotechnology Information (NCBI) website.

[4]The pooling data contain two sources. We sequenced two collections of pooled data containing 30 mallards with ~ 40× coverage, respectively.

[5]The frequency of the mallard allele.

[6]The frequency of the Pekin duck allele.

Supplementary Table 14. Primers used in the experiment in relation to the two critical genes *MITF* and *IGF2BP1*. The putative insertion in *MITF* was examined by PCR, and the primers (*MITF*-gap-F, *MITF*-gap-R) were designed using the flanking sequence of the insertion site. The other 5 primer pairs were designed for RT-qPCR (reverse transcription quantitative PCR).

**Gene**	**Primers**	**Sequence (5′-3′)**	**Annealing temperature**	**Function**
β-actin	b-actin-F	GGTATCGGCAGCAGTCTTA	60℃	qPCR
β-actin	b-actin-R	TTCACAGAGGCGAGTAACTT	60℃	qPCR
MITF	*MITF*-gap-F	AGCTCTTGCTAAGAAGGCTTAC	58℃	genotyping
MITF	*MITF*-gap-R	GGGCTTGCTGGATATGGTA	58℃	genotyping
MITF	*MITF*-exon12-F	GCCAGACACCTGCCATCAAC	60℃	qPCR
MITF	*MITF*-exon12-R	CTGCTTTACCTGCTGCCGC	60℃	qPCR
MITF	*MITF*-exon02-F	TATGTGAATCGCTCAGACTGGAG	60℃	qPCR
MITF	*MITF*-exon02-R	TGGTTGGCGTGTTTATTTGCTA	60℃	qPCR
MITF	*MITF*-exon89-F	AACAGCAACGCACAAAGGA	60℃	qPCR
MITF	*MITF*-exon89-R	GGTGGATGGCACAAGGGAC	60℃	qPCR
IGF2BP1	*IGF2BP1*-F	CATTCCCCTCCGTCTTCTGG	60℃	qPCR
IGF2BP2	*IGF2BP*1-R	TTGGTGTCCTTTGCCTCCTTC	60℃	qPCR

Supplementary Table 15. The top 10 SNPs for body size-related traits in the GWAS based on the F_2 population of 1026.

Trait	Chrom	Pos	-log P value	Additive effect	Additive log P value	Dominance effect	Dominance -log *P* value	Marker R^2
Body weight	28	4411849	31.92	113.310	32.20	9.275	0.26	0.161
	28	4398242	31.26	111.072	31.55	9.344	0.27	0.154
	28	4432053	30.81	106.715	31.51	2.622	0.06	0.151
	28	4504507	30.35	-109.994	31.36	5.332	0.13	0.154
	28	4382810	30.29	-106.108	30.82	42.908	1.98	0.156
	28	4397972	30.07	-108.423	30.71	31.863	1.39	0.154
	28	4420098	30.03	-107.458	31.17	8.383	0.22	0.155
	28	4499383	29.76	-106.349	30.82	19.893	0.70	0.147
	28	4388624	29.50	104.258	29.89	31.613	1.41	0.147
	28	4402545	29.24	-106.332	30.27	24.301	0.89	0.150
Chest width	28	4504507	20.11	-0.208	21.06	0.068	1.11	0.107
	28	4397972	19.84	-0.201	19.86	0.094	1.99	0.108
	28	4432053	19.33	0.194	19.49	0.040	0.53	0.099
	28	4411849	19.30	0.197	18.55	0.066	1.16	0.104
	28	4605725	19.16	-0.210	19.63	-0.011	0.12	0.100
	28	4402545	18.90	-0.197	19.57	0.073	1.27	0.101
	28	4398242	18.81	0.201	19.44	0.009	0.10	0.097
	28	4420098	18.62	-0.196	19.66	0.011	0.11	0.102
	28	4392977	18.39	0.193	18.80	0.032	0.39	0.100
	28	4499824	18.34	-0.193	19.03	0.065	1.15	0.095
Tarsometatarsus length	28	4398242	37.94	3.141	38.65	-0.057	0.05	0.189
	28	4401801	35.68	2.995	36.28	-0.050	0.05	0.177
	28	4499383	35.55	-2.949	36.73	0.276	0.32	0.177
	28	4432053	34.96	2.892	36.04	-0.322	0.37	0.175
	28	4395730	34.57	3.005	35.25	-0.232	0.25	0.183
	28	4397972	34.46	-2.945	35.33	0.655	1.03	0.177
	28	4420098	34.24	-2.893	35.37	0.136	0.13	0.176
	28	4382810	34.09	-2.884	35.25	0.423	0.51	0.177
	28	4411849	34.01	2.997	35.18	-0.583	0.89	0.171
	28	4499824	33.83	-2.890	35.01	0.231	0.26	0.168
Gizzard weight	28	4420098	16.17	-2.978	17.10	-0.081	0.05	0.076
	28	4397972	16.07	-3.003	16.77	0.904	0.88	0.074
	28	4525537	16.00	3.060	17.03	-0.727	0.59	0.075
	28	4408152	15.39	-2.880	16.05	-0.622	0.50	0.072
	28	4401801	15.09	2.936	16.03	-0.366	0.26	0.068
	28	4411849	14.88	2.962	15.75	-0.149	0.10	0.069
	28	4543628	14.71	-2.891	15.72	0.198	0.13	0.067
	28	4392977	14.61	2.843	15.40	0.109	0.06	0.069
	28	4401780	14.53	2.933	15.49	-0.356	0.25	0.066
	28	4382810	14.49	-2.817	15.50	0.357	0.24	0.070
Head weight	28	4398242	40.26	4.821	40.91	-0.045	0.03	0.182
	28	4397972	39.47	-4.725	40.54	0.742	0.70	0.185
	28	4411849	39.04	4.710	38.87	0.540	0.46	0.178

	28	4401801	38.22	4.570	38.12	0.407	0.31	0.172
	28	4420098	37.97	-4.540	39.06	0.987	1.01	0.177
	28	4432053	36.91	4.403	37.55	0.079	0.05	0.169
	28	4543628	36.89	-4.535	37.96	0.620	0.52	0.173
	28	4395730	36.83	4.573	36.81	0.193	0.13	0.176
	28	4392977	36.14	4.439	36.71	0.246	0.16	0.171
	28	4537607	35.94	4.324	36.34	1.167	1.39	0.161
Heart weight	28	4653878	14.33	-0.601	15.13	0.042	0.13	0.077
	28	4729216	14.29	-0.595	14.95	0.000	0.00	0.077
	28	4611631	13.56	-0.594	14.50	0.119	0.46	0.072
	28	4634869	13.54	-0.617	14.39	0.130	0.52	0.072
	28	4411849	13.46	0.544	13.54	0.127	0.55	0.071
	28	4543628	13.13	-0.538	13.97	-0.093	0.34	0.070
	28	4382810	12.94	-0.524	13.64	0.210	1.00	0.072
	28	4397972	12.52	-0.527	13.33	0.135	0.58	0.069
	28	4420098	12.49	-0.520	13.46	0.092	0.34	0.068
	28	4638021	12.39	-0.523	13.36	-0.034	0.10	0.067
Leg weight	28	4398242	35.24	7.011	35.26	0.714	0.38	0.184
	28	4382810	34.95	-6.824	35.76	2.139	1.54	0.191
	28	4392977	34.08	6.784	34.57	0.535	0.24	0.185
	28	4432053	33.45	6.597	34.11	0.142	0.06	0.175
	28	4388624	33.45	6.611	33.96	1.758	1.30	0.175
	28	4402545	33.12	-6.677	33.82	2.201	1.71	0.179
	28	4393013	33.11	-6.618	34.25	1.043	0.57	0.179
	28	4411849	33.11	6.817	33.22	0.654	0.33	0.175
	28	4401801	33.00	6.606	32.50	1.098	0.63	0.170
	28	4504507	32.79	-6.773	33.92	0.821	0.41	0.175
Liver weight	28	4397972	16.14	-2.304	16.78	0.758	1.01	0.085
	28	4420098	16.00	-2.257	16.78	-0.282	0.26	0.085
	28	4382810	15.55	-2.209	16.17	0.937	1.25	0.081
	28	4388624	15.31	2.209	16.16	0.436	0.48	0.078
	28	4402545	15.15	-2.237	16.17	0.221	0.19	0.083
	28	4432053	15.09	2.192	16.02	-0.137	0.11	0.076
	28	4398242	14.93	2.263	15.76	-0.065	0.05	0.075
	28	4401801	14.58	2.212	15.50	-0.259	0.24	0.073
	28	4525537	14.51	2.221	15.51	-0.517	0.54	0.075
	28	4499383	14.10	-2.138	15.07	0.363	0.37	0.070
Wing weight	28	4420098	33.76	-4.432	34.93	0.532	0.41	0.183
	28	4398242	33.66	4.542	34.54	-0.256	0.18	0.174
	28	4411849	33.02	4.501	33.73	-0.030	0.02	0.170
	28	4543628	33.02	-4.431	34.12	-0.301	0.20	0.175
	28	4499824	31.79	-4.284	32.95	0.202	0.14	0.162
	28	4432053	31.63	4.203	32.44	-0.029	0.02	0.162
	28	4392977	31.34	4.295	32.41	-0.414	0.29	0.167
	28	4402545	31.32	-4.260	32.36	0.926	0.89	0.167
	28	4537607	31.18	4.158	31.84	0.944	0.97	0.157
	28	4388624	30.99	4.122	31.28	1.309	1.60	0.160

The statistical table shows the results of the tests for each trait. There is a single line for each marker

tested. The *P* values referenced in the columns are the values of the F distribution for the test of the marker. The columns with “additive” are the *P* values of the F-test on the additive model, and the ones with “dominance” are the *P* values of the F-test of dominance after the additive model has been fitted. The column labeled “Marker R^2” is the R^2 for the marker calculated with a formula for R^2 for a generalized least squares (GLS) model as shown here.

Supplementary Table 16. The top loci of body size-related traits in the genome-wide linkage analysis based on the F_2 population of 1026.

Trait	Genetics position	Bayes interval (95%)	Bayes interval marker (95%)	LOD value	PVE (%)[1]	ADD[2]	PPP (%)[3]
Head weight	4.173	0.045-4.566	chr28_1852078-chr28_4413397	44.05	16.74	0.08	15.59
Wing weight	4.026	0.045-4.566	chr28_1852078-chr28_4413397	33.71	14.10	2.34	15.4
Heart weight	4.173	0.045-6.827	chr28_1852078-chr28_4465718	10.71	5.39	0.55	7.69
Liver weight	3.240	0.045-5.056	chr28_1852078-chr28_4419901	11.94	5.99	2.39	13.27
Leg weight	3.0	0.045-4.173	chr28_1852078-chr28_4402173	37.50	16.14	2.42	19.34
Tarsometatarsus length	3.0	0.045-4.762	chr28_1852078-chr28_4418799	32.37	14.68	3.39	2.9
Chest width	4.811	0.045-6.827	chr28_1852078-chr28_4465718	18.23	8.72	0.21	4.9
Gizzard weight	3.0	0.045-4.811	chr28_1852078-chr28_4419267	11.94	5.66	3.32	13.38
Feed efficiency	8.760	6.827-8.857	chr28_4465718-chr28_4645322	9.33	11.11	-0.13	6.09
Body weight	4.173	0.045-5.547	chr28_1852078-chr28_4420553	29.10	11.38	14.26	15.11

[1]Phenotypic variance explained

[2]Additive effect value

[3]The proportion of phenotypic promotion corresponding to the mallard genotype for a favorable QTL genotype

Supplementary Table 17. Statistics for synonymous and missense variants at the end of chromosome 28 (4,400,000 bp-4,710,000 bp).

Position	Ref[1]	Alt[2]	Variation type	Gene	Ref frequency		
					Mal[3]	Ind[4]	Pek[5]
4519142	G	A	synonymous	*HOXB8*	0.772727	0.972222	1
4533407	C	T	synonymous	*HOXB9*	0.5	0.222222	0
4612138	T	C	missense	*CALCOCO2*	0.693182	0.138889	0
4614069	G	A	missense	*CALCOCO2*	0.151163	0.819444	1
4617824	G	A	missense	*CALCOCO2*	0.715909	0.885714	1
4630498	G	A	synonymous	*UBE2Z*	0.337209	0.916667	1
4643626	G	A	missense	*GIP*	0.863636	0.958333	1

[1]Reference allele

[2]Alternative allele

[3]Mallard

[4]Indigenous breeds

[5]Pekin duck

Supplementary Table 18. Expression levels of 19 protein-coding genes at the end of chromosome 28 in multiple tissues of mallards and Pekin ducks. At 1 day (1D), 2 weeks (2W), 4 weeks (4W), and 8 weeks (8W), tissues were sampled and gene expression levels were measured by RNA-seq. The adjusted values (counts per million) are shown.

**Population**	**Tissue**	**Age**	**Gene**									
			HOXB1	***HOXB2***	***HOXB3***	***HOXB4***	***HOXB5***	***HOXB6***	***HOXB7***	***HOXB8***	***HOXB9***	***HOXB13***
mallard	skin	2W	4	90	122	0	136	360	219	56	60	0
mallard	skin	4W	12	148	294	0	480	1235	491	86	27	0
mallard	skin	8W	6	94	185	0	267	647	304	60	28	0
Pekin	skin	2W	4	103	203	0	327	826	302	65	73	0
Pekin	skin	4W	0	124	203	0	381	883	243	50	53	0
Pekin	skin	8W	4	200	315	0	434	1195	383	70	22	0
mallard	liver	2W	6	34	60	0	6	4	0	0	0	0
mallard	liver	4W	4	13	13	0	14	2	0	0	0	0
mallard	liver	8W	8	19	29	0	30	6	0	0	2	0
Pekin	liver	2W	13	26	51	0	13	0	0	0	0	0
Pekin	liver	4W	7	21	23	0	13	2	0	0	0	0
Pekin	liver	8W	0	13	30	0	22	3	3	0	0	0
mallard	fat	8W	6	136	671	0	422	1981	765	152	188	0
mallard	fat	8W	1	121	1329	0	673	2635	600	161	253	0
mallard	fat	8W	3	163	610	0	387	2102	498	163	199	0
Pekin	fat	8W	1	136	1005	0	649	2529	615	126	105	0
Pekin	fat	8W	3	125	919	0	592	2345	656	142	108	1
Pekin	fat	8W	0	169	745	0	626	2230	698	90	86	0
mallard	knee cartilage	1D	3	7	53	0	32	213	130	119	308	0
mallard	knee cartilage	1D	3	1	27	0	21	149	85	95	283	0

mallard	knee cartilage	1D	2	4	45	0	23	124	52	43	170	0
Pekin	knee cartilage	1D	1	6	51	0	16	110	76	82	217	0
Pekin	knee cartilage	1D	0	3	45	0	25	139	88	190	342	0
Pekin	knee cartilage	1D	3	2	47	0	22	137	59	73	182	0
mallard	breast muscle	1D	21	144	353	0	139	517	269	52	15	0
mallard	breast muscle	2W	10	117	160	0	84	211	103	19	3	0
mallard	breast muscle	2W	14	112	163	0	85	318	141	19	1	0
mallard	breast muscle	4W	2	77	124	0	95	214	188	24	2	0
mallard	breast muscle	4W	11	71	107	0	102	216	188	21	3	0
Pekin	breast muscle	1D	10	240	404	0	73	419	182	31	5	0
Pekin	breast muscle	2W	2	81	261	0	182	528	207	40	14	0
Pekin	breast muscle	2W	7	69	238	0	133	422	185	32	3	0
Pekin	breast muscle	4W	22	155	172	0	170	508	200	63	9	0
Pekin	breast muscle	4W	2	74	166	0	125	338	227	61	12	0
Pekin	breast muscle	8W	4	131	288	0	373	1117	409	76	6	0
mallard	brain	8W	0	0	1	0	0	0	5	0	0	0
Pekin	brain	8W	0	1	1	0	1	3	0	0	1	0
mallard	heart	8W	47	67	84	0	73	15	29	3	1	0
Pekin	heart	8W	33	109	170	0	108	10	7	7	0	0
mallard	lung	8W	22	269	637	0	1032	722	1612	392	2	0
Pekin	lung	8W	7	219	296	0	719	1059	1475	389	2	2
mallard	kidney	8W	379	676	797	0	934	2904	591	872	1229	1
Pekin	kidney	8W	334	738	799	0	1075	3224	758	953	1159	0
mallard	spleen	8W	57	117	129	0	145	64	11	8	0	0
Pekin	spleen	8W	32	163	157	0	185	106	26	2	0	0

Population	Tissue	Stage	Gene								
			CALCOCO2	***ATP5G1***	***UBE2Z***	***SNF8***	***GIP***	***IGF2BP1***	***GNGT2***	***ABI3***	***PHOSPHO1***
mallard	skin	2W	375	2762	466	966	7	38	11	33	666
mallard	skin	4W	819	2307	803	694	9	2	2	119	628
mallard	skin	8W	403	2356	530	780	20	6	8	54	614
Pekin	skin	2W	545	1776	637	812	36	200	5	73	597
Pekin	skin	4W	532	2383	589	876	24	220	7	53	596
Pekin	skin	8W	473	1978	632	742	36	477	15	49	642
mallard	liver	2W	495	8597	553	525	108	6	4	1049	1621
mallard	liver	4W	899	10521	709	548	34	0	23	728	1706
mallard	liver	8W	1028	6746	771	822	68	4	0	1211	997
Pekin	liver	2W	565	10581	573	490	72	255	11	872	1704
Pekin	liver	4W	668	8471	475	477	87	48	12	282	2112
Pekin	liver	8W	783	7471	778	592	96	22	3	759	1161
mallard	fat	8W	1207	748	525	321	22	0	2	76	648
mallard	fat	8W	1018	766	529	320	6	0	0	58	473
mallard	fat	8W	1131	869	503	326	36	0	1	37	478
Pekin	fat	8W	1381	916	619	366	113	44	5	27	349
Pekin	fat	8W	1336	604	536	286	89	43	5	41	315
Pekin	fat	8W	1569	583	578	314	59	42	8	163	353
mallard	knee cartilage	1D	1226	2469	645	306	57	37	50	144	13628
mallard	knee cartilage	1D	881	2717	382	211	22	49	8	43	7531
mallard	knee cartilage	1D	846	1549	396	197	26	25	8	62	7177
Pekin	knee cartilage	1D	615	2283	261	238	53	194	13	51	6754
Pekin	knee cartilage	1D	1224	2859	473	391	85	222	28	45	11240
Pekin	knee cartilage	1D	810	2316	374	267	82	192	30	71	6491

mallard	breast muscle	1D	497	7646	667	1026	54	269	11	33	1738
mallard	breast muscle	2W	321	4988	709	569	18	195	16	31	501
mallard	breast muscle	2W	381	8110	634	665	31	193	4	38	872
mallard	breast muscle	4W	470	9243	561	515	17	49	7	84	687
mallard	breast muscle	4W	321	15652	444	524	45	148	8	30	593
Pekin	breast muscle	1D	453	4916	703	724	74	414	14	13	1210
Pekin	breast muscle	2W	603	6197	715	526	38	177	5	54	578
Pekin	breast muscle	2W	477	9013	675	614	34	248	6	33	806
Pekin	breast muscle	4W	548	12031	595	498	33	105	2	103	596
Pekin	breast muscle	4W	622	19926	594	865	35	78	0	76	746
Pekin	breast muscle	8W	549	13292	701	565	37	15	1	119	2315
mallard	brain	8W	242	2227	581	905	14	1	5	92	1074
Pekin	brain	8W	288	2135	694	857	53	12	4	106	1141
mallard	heart	8W	613	16984	897	655	9	1	3	132	960
Pekin	heart	8W	725	21189	808	1043	24	10	2	85	824
mallard	lung	8W	652	1027	656	738	15	24	10	268	608
Pekin	lung	8W	732	846	919	741	57	138	12	397	925
mallard	kidney	8W	706	4193	678	810	84	0	8	192	1631
Pekin	kidney	8W	744	4046	666	673	165	33	6	179	1530
mallard	spleen	8W	880	1685	614	810	9	9	65	8227	704
Pekin	spleen	8W	882	1402	682	854	21	119	115	7508	671

Supplementary Table 19. Expression levels of *IGF2BP1* in multiple tissues of mallards and Pekin ducks. Tissues from 1-day-old (1D), 2-week-old (2W), 4-week-old (4W) and 8-week-old (8W) ducks were sampled, and the gene expression levels were measured by qPCR.

Tissue	Population	Developmental stage	$2^{-\Delta\Delta Ct}$	Average of $2^{-\Delta\Delta Ct}$	SD of $2^{-\Delta\Delta Ct}$
brain	mallard	1D	0.169893081	0.139180483	0.049021605
brain	mallard	1D	0.165002697		
brain	mallard	1D	0.08264567		
brain	mallard	2W	0.047394365	0.056635786	0.008298256
brain	mallard	2W	0.059063745		
brain	mallard	2W	0.063449247		
brain	mallard	4W	0.028165165	0.029837935	0.003937097
brain	mallard	4W	0.02701343		
brain	mallard	4W	0.03433521		
brain	mallard	8W	0.010857966	0.010498915	0.004890556
brain	mallard	8W	0.005438729		
brain	mallard	8W	0.01520005		
brain	Pekin duck	1D	1.367283899	1.344239917	0.076290462
brain	Pekin duck	1D	1.406351924		
brain	Pekin duck	1D	1.259083927		
brain	Pekin duck	2W	0.195582101	0.183885181	0.010193625
brain	Pekin duck	2W	0.179175387		
brain	Pekin duck	2W	0.176898056		
brain	Pekin duck	4W	0.063877647	0.055834764	0.00703385
brain	Pekin duck	4W	0.050834002		
brain	Pekin duck	4W	0.052792642		
brain	Pekin duck	8W	0.018106522	0.037152532	0.022086379
brain	Pekin duck	8W	0.061363807		
brain	Pekin duck	8W	0.031987267		
breast muscle	mallard	1D	2.45099174	2.323543738	0.189319784
breast muscle	mallard	1D	2.413636952		
breast muscle	mallard	1D	2.106002521		
breast muscle	mallard	2W	1.740244906	1.692724105	0.044267132
breast muscle	mallard	2W	1.685270425		
breast muscle	mallard	2W	1.652656985		
breast muscle	mallard	4W	0.214117663	0.206110883	0.042204072
breast muscle	mallard	4W	0.160476948		
breast muscle	mallard	4W	0.243738037		
breast muscle	mallard	8W	0.002316255	0.002686569	0.000353748
breast muscle	mallard	8W	0.003021019		
breast muscle	mallard	8W	0.002722432		
breast muscle	Pekin duck	1D	2.635994171	2.375822856	1.494602805
breast muscle	Pekin duck	1D	3.723259013		

breast muscle	Pekin duck	1D	0.768215385		
breast muscle	Pekin duck	2W	1.413111758		
breast muscle	Pekin duck	2W	1.478677746	1.421857476	0.052991478
breast muscle	Pekin duck	2W	1.373782922		
breast muscle	Pekin duck	4W	0.258923276		
breast muscle	Pekin duck	4W	0.207375156	0.240023132	0.028391096
breast muscle	Pekin duck	4W	0.253770964		
breast muscle	Pekin duck	8W	0.03551796		
breast muscle	Pekin duck	8W	0.021464709	0.027039138	0.007463249
breast muscle	Pekin duck	8W	0.024134744		
heart	mallard	1D	3.07701824		
heart	mallard	1D	3.767082633	3.447186216	0.347768082
heart	mallard	1D	3.497457775		
heart	mallard	2W	0.830434707		
heart	mallard	2W	1.149645396	0.971942451	0.162654343
heart	mallard	2W	0.935747251		
heart	mallard	4W	0.031803418		
heart	mallard	4W	0.031666706	0.03178764	0.000113868
heart	mallard	4W	0.031892795		
heart	mallard	8W	0.001980154		
heart	mallard	8W	0.002263147	0.001875015	0.000450009
heart	mallard	8W	0.001381744		
heart	Pekin duck	1D	7.560585842		
heart	Pekin duck	1D	6.962261146	6.829096608	0.806360786
heart	Pekin duck	1D	5.964442837		
heart	Pekin duck	2W	1.538082653		
heart	Pekin duck	2W	4.083710433	2.628368733	1.311489384
heart	Pekin duck	2W	2.263313114		
heart	Pekin duck	4W	1.313644924		
heart	Pekin duck	4W	1.092917503	1.223989192	0.116045723
heart	Pekin duck	4W	1.26540515		
heart	Pekin duck	8W	0.188138782		
heart	Pekin duck	8W	0.056169543	0.121096374	0.06601005
heart	Pekin duck	8W	0.118980798		
kidney	mallard	1D	0.185319782		
kidney	mallard	1D	0.248560461	0.221270522	0.032497738
kidney	mallard	1D	0.229931323		
kidney	mallard	2W	0.023225064		
kidney	mallard	2W	0.017803259	0.020161104	0.002779018
kidney	mallard	2W	0.019454987		
kidney	mallard	4W	0.015667312		
kidney	mallard	4W	0.015619161	0.015376045	0.000463414
kidney	mallard	4W	0.014841664		
kidney	mallard	8W	0.003945052		
kidney	mallard	8W	0.005867351	0.004639946	0.00106606
kidney	mallard	8W	0.004107435		
kidney	Pekin duck	1D	6.856619444	5.572080088	1.264907374
kidney	Pekin duck	1D	5.531856628		

kidney	Pekin duck	1D	4.327764193		
kidney	Pekin duck	2W	1.809607367	2.24984889	0.608920011
kidney	Pekin duck	2W	2.944758163		
kidney	Pekin duck	2W	1.995181141		
kidney	Pekin duck	4W	0.496152011	0.540243817	0.054524974
kidney	Pekin duck	4W	0.601211529		
kidney	Pekin duck	4W	0.523367912		
kidney	Pekin duck	8W	0.026078511	0.025706944	0.016523148
kidney	Pekin duck	8W	0.042041175		
kidney	Pekin duck	8W	0.009001147		
liver	mallard	1D	2.95300015	2.999628896	0.052779955
liver	mallard	1D	2.988957336		
liver	mallard	1D	3.056929202		
liver	mallard	2W	0.096183534	0.098681884	0.002439993
liver	mallard	2W	0.098803116		
liver	mallard	2W	0.101059001		
liver	mallard	4W	0.016505097	0.01777328	0.001113489
liver	mallard	4W	0.018223954		
liver	mallard	4W	0.018590788		
liver	mallard	8W	0.007444676	0.008172379	0.00088425
liver	mallard	8W	0.0091565		
liver	mallard	8W	0.007915962		
liver	Pekin duck	1D	8.23822086	6.631290926	1.447691723
liver	Pekin duck	1D	5.428898064		
liver	Pekin duck	1D	6.226753855		
liver	Pekin duck	2W	5.704948814	4.532161383	1.247473767
liver	Pekin duck	2W	4.670072873		
liver	Pekin duck	2W	3.221462462		
liver	Pekin duck	4W	2.917470022	2.435055759	1.140092333
liver	Pekin duck	4W	1.13306218		
liver	Pekin duck	4W	3.254635075		
liver	Pekin duck	8W	1.860769755	1.542526343	0.300647504
liver	Pekin duck	8W	1.503528615		
liver	Pekin duck	8W	1.263280659		
lung	mallard	1D	1.055836192	0.946154358	0.096073171
lung	mallard	1D	0.905717462		
lung	mallard	1D	0.876909422		
lung	mallard	2W	0.123241905	0.139956093	0.014815497
lung	mallard	2W	0.145154724		
lung	mallard	2W	0.15147165		
lung	mallard	4W	0.008309554	0.009048814	0.002516513
lung	mallard	4W	0.006984732		
lung	mallard	4W	0.011852157		
lung	mallard	8W	0.004316315	0.005144349	0.00081458
lung	mallard	8W	0.005944774		
lung	mallard	8W	0.005171959		
lung	Pekin duck	1D	4.147385719	4.151711404	0.409059052
lung	Pekin duck	1D	4.562916144		

lung	Pekin duck	1D	3.744832348		
lung	Pekin duck	2W	3.336004559	2.847867167	0.762114506
lung	Pekin duck	2W	3.237919859		
lung	Pekin duck	2W	1.969677083		
lung	Pekin duck	4W	0.534133767	0.566499111	0.031005862
lung	Pekin duck	4W	0.569425583		
lung	Pekin duck	4W	0.595937983		
lung	Pekin duck	8W	0.246191058	0.175581632	0.073250598
lung	Pekin duck	8W	0.099948495		
lung	Pekin duck	8W	0.180605342		
skin	mallard	1D	0.105937547	0.068467819	0.032509733
skin	mallard	1D	0.047758784		
skin	mallard	1D	0.051707124		
skin	mallard	2W	0.040188711	0.037214623	0.003248003
skin	mallard	2W	0.033748785		
skin	mallard	2W	0.037706373		
skin	mallard	4W	0.019647042	0.017633589	0.007111707
skin	mallard	4W	0.009732236		
skin	mallard	4W	0.02352149		
skin	mallard	8W	0.003542538	0.003615194	7.72761E-05
skin	mallard	8W	0.003606661		
skin	mallard	8W	0.003696382		
skin	Pekin duck	1D	5.402232126	5.242504785	0.880544907
skin	Pekin duck	1D	6.032252934		
skin	Pekin duck	1D	4.293029296		
skin	Pekin duck	2W	2.814602397	3.241444881	0.681003923
skin	Pekin duck	2W	4.026810581		
skin	Pekin duck	2W	2.882921664		
skin	Pekin duck	4W	0.44075783	0.403298296	0.038947498
skin	Pekin duck	4W	0.406120676		
skin	Pekin duck	4W	0.363016382		
skin	Pekin duck	8W	0.059633529	0.068366372	0.008239911
skin	Pekin duck	8W	0.069461827		
skin	Pekin duck	8W	0.07600376		
spleen	mallard	1D	0.031439455	0.037692673	0.01334221
spleen	mallard	1D	0.053013031		
spleen	mallard	1D	0.028625534		
spleen	mallard	2W	0.011757004	0.012923204	0.003243648
spleen	mallard	2W	0.016588711		
spleen	mallard	2W	0.010423896		
spleen	mallard	4W	0.002261035	0.002269777	7.77434E-06
spleen	mallard	4W	0.002275914		
spleen	mallard	4W	0.002272383		
spleen	mallard	8W	0.006444569	0.008095018	0.001659281
spleen	mallard	8W	0.008077494		
spleen	mallard	8W	0.009762993		
spleen	Pekin duck	1D	2.650373885	2.133002517	0.699428849
spleen	Pekin duck	1D	1.337245269		

spleen	Pekin duck	1D	2.411388396		
spleen	Pekin duck	2W	1.681342377	1.722936457	0.036148383
spleen	Pekin duck	2W	1.740707772		
spleen	Pekin duck	2W	1.746759221		
spleen	Pekin duck	4W	1.064177141	1.065961458	0.003295307
spleen	Pekin duck	4W	1.063943083		
spleen	Pekin duck	4W	1.06976415		
spleen	Pekin duck	8W	0.202661643	0.204004244	0.022304552
spleen	Pekin duck	8W	0.182401319		
spleen	Pekin duck	8W	0.226949769		

Supplementary Table 20. Expression levels of *IGF2BP1* in different recombinant types of F_2 individuals. The gene expression levels were measured by qPCR.

Sample name	Recombinant type	$2^{-\Delta\Delta Ct}$
895	P	0.839464178
431	P	1.477196385
990	P	1.728455265
678	P	2.216168153
717	P	2.651327034
866	P	3.296683006
918L	P	3.873307685
88	P	4.017512164
668	P	4.056285726
51	P	5.112021104
665	P	5.311701846
933L	P	6.333848312
69L	P	6.692382437
98L	P	7.917629261
746L	P	7.987929967
103	H	0.494264679
540	H	0.66184664
832	H	0.729604511
446	H	0.821382435
639	H	0.910401566
373	H	0.921942575
535	H	0.999615706
124	H	1.195338171
820	H	1.268893147
55	H	1.347411279
801	H	1.445646953
390	H	1.454321286
333	H	1.769664693
227	H	1.929074571
718	H	2.045556615
729L	M	0.000349275
550	M	0.002723203
204	M	0.00298755
554L	M	0.006166086
902	M	0.007911596
689	M	0.008625992
396	M	0.017566253
180	M	0.018882645
161	M	0.02551996
537	M	0.028679569
657L	M	0.031951006

911	M	0.034613187
666	M	0.040262044
232L	M	0.059194811
651	M	0.063339075
230	R1	0.007874472
695L	R1	0.009174732
489	R1	0.010446862
322	R1	0.011839868
253	R1	0.01400941
297	R1	0.018722055
930	R1	0.019336488
906	R1	0.022076956
201	R1	0.024366476
182	R1	0.025965949
448	R1	0.027232169
257	R1	0.030498238
352	R1	0.032096054
66	R1	0.035449446
398	R1	0.041210537
983	R1	0.046246013
541	R1	0.05069314
640	R1	0.064015123
458	R1	0.078939527
409	R1	0.085548977
454	R1	0.091746603
680	R1	0.097519559
358	R2	0.026406705
940	R2	0.063443
620	R3	0.403338502
712	R3	0.433021876
175	R3	0.470098434
658	R3	0.609334566
225	R3	0.749452646
786	R4	0.53967138
850	R4	0.596306988
395	R4	0.8067074
860	R4	1.074101415
311	R4	1.441377165
504	R4	1.482118218
255	R4	1.807245756
122	R5	0.362255903
132	R5	0.393219641
415	R5	0.456165701
68L	R5	0.466018865
782L	R5	0.547255966
197	R5	0.715291098

955L	R5	0.743387997
450	R5	0.85268732
85	R5	0.884012687
480L	R5	1.005095785
663	R5	1.129129497
494L	R5	1.145232943
252L	R5	1.276825905
996L	R5	1.888621343
888L	R5	2.644543722
950L	R5	2.974070742
5	R5	2.978646011
244L	R5	3.08052137
635L	R5	3.19692428
403L	R5	3.362504948
699	R6	0.487087052
691	R6	0.53496058
490	R6	0.855998834
338	R6	0.910770349
385	R6	1.591251724
194	R6	2.619883608
595	R6	3.028837747
43	R6	3.125893331
807	R7	1.114457368
702	R7	1.527563797
96	R7	1.651033593
845	R7	2.061801967
736	R7	2.576552224
486	R7	3.10639315
707	R7	3.182544121
572	R7	3.323153013
237	R7	3.473506875
818	R7	3.666052383
129	R7	4.08048624
168	R7	4.155867305

Supplementary Table 21. Summary of Hi-C data in duck. This table shows the number of Hi-C sequencing reads in the category of each processing step in HiC-Pro.

	Read count
Total read pairs	1,063,446,129
Uniquely aligned read pairs	554,436,799
Dumped read pairs	16,248,309
Self-circle	824,009
Dangling-end	155,542,662
Valid interactions	347,550,446
Filtered valid interactions	129,168,069
Intrachromosomal contacts	71,680,763
Interchromosomal contacts	57,487,306

Supplementary Table 22. ***IGF2BP1*** **expression levels** in (**a**) human, (**b**) mouse, (**c**) chicken, and (**d**) zebrafish.

a

Accession	Tissue	Developmental stage	RPKM
SAMN03267750	heart	embryonic (10.4 weeks)	12.701
SAMN03267763	heart	embryonic (11.4 weeks)	4.802
SAMN03267757	heart	embryonic (17.5 weeks)	2.527
SAMN03267751	heart	embryonic (18 weeks)	2.295
SAMN03267764	heart	embryonic (20 weeks)	2.218
SAMN03267760	heart	embryonic (20 weeks)	1.616
SAMN03267756	heart	embryonic (20.3 weeks)	1.015
SAMN03267784	kidney	embryonic (10.3 weeks)	10.181
SAMN03267772	kidney	embryonic (16.4 weeks)	1.7
SAMN03267777	kidney	embryonic (20 weeks)	0.176
SAMN03267770	lung	embryonic (10.4 weeks)	9.148
SAMN03267767	lung	embryonic (10.2 weeks)	10.545
SAMN03267778	lung	embryonic (17.2 weeks)	2.159
SAMN03267776	lung	embryonic (17.5 weeks)	2.888
SAMN03267768	lung	embryonic (20 weeks)	2.574
SAMN03267780	lung	embryonic (20.6 weeks)	1.045
SAMN03267754	adrenal	embryonic (10.3 weeks)	5.907
SAMN03267755	adrenal	embryonic (16.4 weeks)	3.329
SAMN03267761	adrenal	embryonic (18 weeks)	2.808
SAMN03267753	adrenal	embryonic (20.2 weeks)	2.561
SAMN03267762	adrenal	embryonic (20.6 weeks)	1.302
SAMN03267779	stomach	embryonic (10.3 weeks)	5.847
SAMN03267773	stomach	embryonic (10.4 weeks)	5.732
SAMN03267774	stomach	embryonic (16.4 weeks)	2.096
SAMN03267771	stomach	embryonic (18 weeks)	1.757
SAMN03267783	stomach	embryonic (20 weeks)	1.009
SAMN03267781	stomach	embryonic (20.3 weeks)	1.743
SAMN03267765	stomach	embryonic (20.6 weeks)	1.003
SAMN03267752	intestine	embryonic (10.4 weeks)	5.127
SAMN03267759	intestine	embryonic (10.3 weeks)	4.251
SAMN03267758	intestine	embryonic (11.5 weeks)	8.091
SAMN03267775	intestine	embryonic (15.3 weeks)	5.467
SAMN03267782	intestine	embryonic (17.1 weeks)	2.225
SAMN03267769	intestine	embryonic (20 weeks)	4.028
SAMN03267766	intestine	embryonic (20.6 weeks)	4.52
SAMEA2145743	brain	adult	0.004
SAMEA2154665	brain	adult	0
SAMEA2157437	brain	adult	0
SAMEA2151741	heart	adult	0
SAMEA2154361	heart	adult	0

SAMEA2155550	heart	adult	0.005
SAMEA2159607	heart	adult	0.01
SAMEA1970526	kidney	adult	1.039
SAMEA2145774	kidney	adult	0.257
SAMEA2159080	kidney	adult	0.522
SAMEA2162328	kidney	adult	1.118
SAMEA2145122	liver	adult	0.013
SAMEA2155590	liver	adult	0.021
SAMEA2162895	liver	adult	0.018
SAMEA2142363	lung	adult	0.477
SAMEA2144333	lung	adult	0.012
SAMEA2147920	lung	adult	0.002
SAMEA2155770	lung	adult	0.001
SAMEA2158569	lung	adult	0
SAMEA2145893	skin	adult	0
SAMEA2147596	skin	adult	0
SAMEA2163623	skin	adult	0
SAMEA2146236	spleen	adult	0.014
SAMEA2153031	spleen	adult	0.014
SAMEA2155751	spleen	adult	0.003
SAMEA2159764	spleen	adult	0.037
SAMEA2145544	adrenal	adult	0
SAMEA2146411	adrenal	adult	0.006
SAMEA2162841	adrenal	adult	0.005
SAMEA1966682	appendix	adult	0.063
SAMEA2142670	appendix	adult	0.019
SAMEA2151358	appendix	adult	0.017
SAMEA2149004	bone marrow	adult	0
SAMEA2154529	bone marrow	adult	0
SAMEA2162823	bone marrow	adult	0
SAMEA2163105	bone marrow	adult	0
SAMEA1968540	colon	adult	0.004
SAMEA2142348	colon	adult	0.006
SAMEA2152568	colon	adult	0.011
SAMEA2152798	colon	adult	0.002
SAMEA2156266	colon	adult	0
SAMEA2144835	duodenum	adult	0.003
SAMEA2153946	duodenum	adult	0.033
SAMEA1968189	endometrium	adult	0
SAMEA2155974	endometrium	adult	0
SAMEA2156099	endometrium	adult	0.04
SAMEA2147975	esophagus	adult	0
SAMEA2156670	esophagus	adult	0
SAMEA2158800	esophagus	adult	0.002
SAMEA2142680	fat	adult	0.001

SAMEA2153980	fat	adult	0.013
SAMEA2154125	fat	adult	0
SAMEA2146982	gall bladder	adult	0.002
SAMEA2159912	gall bladder	adult	0.016
SAMEA2163459	gall bladder	adult	0.032
SAMEA1965299	lymph node	adult	0.303
SAMEA2149876	lymph node	adult	0.189
SAMEA2150385	lymph node	adult	0.11
SAMEA2152719	lymph node	adult	0.072
SAMEA2155628	lymph node	adult	0
SAMEA2151405	pancreas	adult	0.001
SAMEA2153347	pancreas	adult	0
SAMEA2153427	prostate	adult	0.005
SAMEA2155984	prostate	adult	0.005
SAMEA2159368	prostate	adult	0
SAMEA2162946	prostate	adult	0
SAMEA1968832	salivary gland	adult	0.001
SAMEA2150585	salivary gland	adult	0
SAMEA2151887	salivary gland	adult	0.002
SAMEA2152474	small intestine	adult	0.056
SAMEA2153733	small intestine	adult	0.015
SAMEA2153932	small intestine	adult	0
SAMEA2161836	small intestine	adult	0.018
SAMEA2142586	stomach	adult	0.018
SAMEA2154405	stomach	adult	0.009
SAMEA2157511	stomach	adult	0.009
SAMEA2144120	thyroid	adult	0
SAMEA2145240	thyroid	adult	0.003
SAMEA2145313	thyroid	adult	0.002
SAMEA2159931	thyroid	adult	0
SAMEA2144335	urinary bladder	adult	0.01
SAMEA2161920	urinary bladder	adult	0
SAMEA2145245	ovary	adult	0.003
SAMEA2155371	ovary	adult	0.003
SAMEA2142853	placenta	adult	3.622
SAMEA2149398	placenta	adult	2.551
SAMEA2153307	placenta	adult	2.805
SAMEA2162568	placenta	adult	2.533
SAMEA1968968	testis	adult	3.261
SAMEA2148093	testis	adult	2.754
SAMEA2148874	testis	adult	4.045
SAMEA2149178	testis	adult	4.825
SAMEA2151119	testis	adult	3.211
SAMEA2158188	testis	adult	3.553
SAMEA2161674	testis	adult	3.804

b

Accession	Tissue	Developmental stage	RPKM
SAMN01164143	liver	embryonic day 14	3.198
SAMN01164140	liver	embryonic day 14.5	2.708
SAMN01164135	liver	embryonic day 18	0.753
SAMN01164142	central nervous system (CNS)	embryonic day 11.5	17.896
SAMN01164138	CNS	embryonic day 14	4.293
SAMN01164139	CNS	embryonic day 18	1.387
SAMN01164141	whole brain	embryonic day 14.5	3.497
SAMN01164137	limb	embryonic day 14.5	7.249
SAMN00849379	adrenal	adult (8 weeks)	0.018
SAMN01164133	bladder	adult (8 weeks)	0.147
SAMN01164136	cerebellum	adult (8 weeks)	0.001
SAMN00849389	colon	adult (8 weeks)	0.101
SAMN01164132	cortex	adult (8 weeks)	0.004
SAMN00849378	duodenum	adult (8 weeks)	0.004
SAMN01164131	frontal lobe	adult (8 weeks)	0.017
SAMN00849381	genital fat pad	adult (8 weeks)	0.009
SAMN00849390	heart	adult (8 weeks)	0.005
SAMN00849385	kidney	adult (8 weeks)	0.155
SAMN00849380	large intestine	adult (8 weeks)	0.003
SAMN00849386	liver	adult (8 weeks)	0
SAMN00849387	lung	adult (8 weeks)	0.241
SAMN00849375	mammary gland	adult (8 weeks)	0.01
SAMN00849377	small intestine	adult (8 weeks)	0.001
SAMN00849388	spleen	adult (8 weeks)	0.003
SAMN00849376	stomach	adult (8 weeks)	0.001
SAMN00849382	subcutaneous fat pad	adult (8 weeks)	0.011
SAMN00849383	thymus	adult (8 weeks)	0.006
SAMN00849374	ovary	adult (8 weeks)	0.011
SAMN01164134	placenta	adult (8 weeks)	10.303
SAMN00849384	testis	adult (8 weeks)	1.029

c

Accession	Tissue	Developmental stage	RPKM
SAMEA3109051	embryonic cell	embryonic stage	27.0434
SAMN02729306	cochlea	embryonic day 6.5	13.1688
SAMN02729309	cochlea	embryonic day 6.5	14.817
SAMN02729314	cochlea	embryonic day 6.5	15.7731
SAMN02729317	cochlea	embryonic day 6.5	33.5247
SAMN02729316	cochlea	embryonic day 6.5	44.6274
SAMN00632119	brain	1 year	0
SAMN00632120	brain	1 year	0
SAMEA2201367	female cerebrum	20 months	0.016194
SAMEA2201363	male cerebrum	20 months	0.0508896

SAMEA2201366	female cerebellum	20 months	0
SAMEA2201381	male cerebellum	20 months	0.0614794
SAMN00632121	cerebellum	1 year	0
SAMN00632122	cerebellum	1 year	0
SAMEA2201377	hypothalamus	20 months	0.018766
SAMEA2201368	hypothalamus	20 months	0.0241758
SAMEA2201361	female parietal nerve	20 months	0.0894499
SAMEA2201383	male parietal nerve	20 months	0.182875
SAMEA2201358	female adipose	20 months	0
SAMEA2201374	male adipose	20 months	0.00790446
SAMEA2201369	breast muscle	20 months	0.0241766
SAMEA2201373	breast muscle	20 months	0.00890807
SAMN04013381	breast muscle	7 weeks	4.1617
SAMN04013382	breast muscle	7 weeks	4.7383
SAMN04013383	breast muscle	7 weeks	0
SAMN04013384	breast muscle	7 weeks	1.35757
SAMEA2201362	lung	20 months	0.0190786
SAMEA2201357	lung	20 months	0.0264387
SAMEA2201379	female heart	20 months	0.0223777
SAMEA2201382	male heart	20 months	0.0246378
SAMN00632123	heart	1 year	0.0294984
SAMN00632124	heart	1 year	0
SAMEA2201378	liver	20 months	0.0102974
SAMEA2201370	liver	20 months	0.0101334
SAMN00632127	liver	1 year	0
SAMN00632128	liver	1 year	0
SAMEA2201371	female proventriculus	20 months	0.446581
SAMEA2201359	male proventriculus	20 months	0.699748
SAMN03376186	duodenum	61 weeks	0.0332268
SAMN03376187	duodenum	61 weeks	0.129813
SAMN03376188	duodenum	61 weeks	0.17816
SAMN03376183	duodenum	61 weeks	0.147607
SAMN03376184	duodenum	61 weeks	0.103226
SAMN03376185	duodenum	61 weeks	0.0425381
SAMEA2201375	adrenal gland	20 months	0.105851
SAMEA2201376	female kidney	20 months	0.0489427
SAMEA2201372	male kidney	20 months	0.0549427
SAMN00632125	kidney	1 year	0.122893
SAMN00632126	kidney	1 year	0.0705325
SAMEA2201365	female spleen	20 months	0.0362605
SAMEA2201380	male spleen	20 months	0.213325
SAMEA2201364	ovary	20 months	49.0721
SAMEA2201360	testis	20 months	0.57776
SAMN00632129	testis	1 year	1.70657
SAMN00632130	testis	1 year	1.70641

d

Accession	Tissue	Developmental stage	RPKM
SAMN02418766	liver	5 dpf	2.32619
SAMN02418767	liver	5 dpf	1.56098
SAMN02418768	liver	5 dpf	2.94774
SAMN02418769	liver	5 dpf	7.31549
SAMEA898401	embryo	1 dpf	35.7963
SAMEA898400	embryo	2 cells	10.2887
SAMEA898403	embryo	2 dpf	27.2926
SAMEA898404	embryo	3 dpf	13.7009
SAMEA708828	embryo	5 dpf	13.7668
SAMEA898399	embryo	6 dpf	12.4668
SAMN02259096	anterior pectoral fin replicate	6 months	0.421936
SAMN02259097	anterior pectoral fin replicate	6 months	0.399351
SAMN02259098	posterior pectoral fin replicate	6 months	0.60389
SAMN02259099	posterior pectoral fin replicate	6 months	0.485261
SAMN02951651	blood	adult	0
SAMN02951648	brain	adult	0.116579
SAMEA708836	head	adult	0.257205
SAMEA708835	head	adult	0
SAMN02951650	heart	adult	0
SAMN02951649	liver	adult	0.0319476
SAMN02951652	muscle	adult	0
SAMN01765705	pineal gland	adult	0.595387
SAMEA708832	ovary	adult	0
